# The Eastern Tropical Pacific coral population connectivity and the role of the Eastern Pacific Barrier

**DOI:** 10.1038/s41598-018-27644-2

**Published:** 2018-06-19

**Authors:** Mauricio Romero-Torres, Eric A. Treml, Alberto Acosta, David A. Paz-García

**Affiliations:** 1 Unidad de Ecología y Sistemática (UNESIS), Departamento de Biología, Pontificia Universidad Javeriana, Carrera 7 No. 40 – 62, Bogotá, Colombia; 2 Deakin University, School of Life and Environmental Sciences, Waurn Ponds, VIC 3216 Australia; 3 School of BioSciences, University of Melbourne, Parkville, Victoria 3010 Australia; 4 Marine Speciation and Molecular Evolution Laboratory. Department of Biological Sciences, Louisiana State University, Baton Rouge, LA 70803 USA; 5 Laboratorio de Necton y Ecología de Arrecifes, Centro de Investigaciones Biológicas del Noroeste (CIBNOR), Calle IPN 195, Col. Playa Palo de Santa Rita Sur, 23096 La Paz, B.C.S. Mexico

## Abstract

Long-distance dispersal is believed to strongly influence coral reef population dynamics across the Tropical Pacific. However, the spatial scale and strength at which populations are potentially connected by dispersal remains uncertain. To determine the patterns in connectivity between the Eastern (ETP) and Central Tropical Pacific (CTP) ecoregions, we used a biophysical model incorporating ocean currents and larval biology to quantify the seascape-wide dispersal potential among all population. We quantified the likelihood and determined the oceanographic conditions that enable the dispersal of coral larvae across the Eastern Pacific Barrier (EP-Barrier) and identified the main connectivity pathways and their conservation value for dominant reef-building corals. Overall, we found that coral assemblages within the CTP and ETP are weakly connected through dispersal. Although the EP-Barrier isolates the ETP from the CTP ecoregion, we found evidence that the EP-Barrier may be breached, in both directions, by rare dispersal events. These rare events could explain the evolutionary genetic similarity among populations of pocilloporids in the ecoregions. Moreover, the ETP may function as a stronger source rather than a destination, providing potential recruits to CTP populations. We also show evidence for a connectivity loop in the ETP, which may positively influence long-term population persistence in the region. Coral conservation and management communities should consider eight-key stepping stone ecoregions when developing strategies to preserve the long-distance connectivity potential across the ETP and CTP.

## Introduction

Coral reefs are one of the most diverse ecosystems in the world, harbouring thousands of species and providing essential ecosystem services to coastal economies and livelihoods. However, anthropogenic disturbances and global warming have reduced coral populations worldwide^1^. Understanding the connectivity (i.e., the dispersal movements) between coral populations is critical in predicting how marine populations and reef ecosystems will cope with climate change and developing effective management and conservation efforts to sustain healthy coral reef communities^2,3^.

Eastern Tropical Pacific (ETP) coral assemblages (Anthozoa: Scleractinia) are unique; they experience some of the most severe environmental stresses endured by reef corals anywhere in the world^4^. These stresses include high-pCO_2_ concentrations, low aragonite saturation, and high levels of nutrients^5^, as well as regions of high tidal amplitude and extreme warm and cold El Niño-Southern Oscillation (ENSO) events. Besides enduring harsh environmental conditions, coral populations in the ETP are geographically isolated from the Central Tropical Pacific (CTP) by a stretch of ~5000 km of open ocean, known as the Eastern Pacific Barrier (EP-Barrier)^6^. For 65 Myr, the EP-Barrier has likely impeded the transpacific dispersal of organisms with planktonic life stages^7^. Yet the co-occurrence of corals species and other marine organisms in both the CTP and ETP has led to the development of several biogeographic hypotheses to explain their origin; these involve historical colonisation dynamics and rare long-distance dispersal across the EP-Barrier^8–10^.

The origin of scleractinian corals in the ETP is believed to have occurred during three mutually non-exclusive periods of colonisation^11^. The current coral reef populations in the ETP are believed to be remnants of Caribbean reef communities which were separated by the closure of the Panama Isthmus during the early Pliocene and influenced by present-day immigration from reefs in the CTP via the North Equatorial Counter Current (NECC). Three hypotheses have been suggested to explain the regular or occasional west-to-east breaching of the EP-Barrier. The first assumes that some species are well adapted for long-distance dispersal and can disperse eastward from the Line Islands via the NECC^6,12–14^. The second predicts that the Clipperton Atoll is a stepping-stone providing a pathway from the CTP to the American continental reef communities^10,15^. The last hypothesis suggests that breaching the EP-Barrier may be possible during intense El Niño events when the NECC accelerates its eastward flow and reduces the transport time required to travel from the Line Islands to the Clipperton Atoll and into the ETP^14,16–18^. This last hypothesis is supported by sporadic observations of Central Pacific fish, molluscs, and sea urchins in the ETP after strong El Niño events^15,19,20^. Yet recent population genetic studies and biophysical models indicate that most of the ETP coral populations have evolved independently from CTP populations^20,21^. For example, ETP populations of *Porites lobata* have been isolated from those in the CTP for thousands of years^11,22^. However, there is evidence of an evolutionarily-significant, and likely ongoing, transpacific gene flow in the dominant reef-building genus *Pocillopora*^23,24^.

ENSO events not only influence transpacific dispersal by accelerating the NECC eastward flow, but it is hypothesised that they also disrupt the coral’s reproductive activities by thermal stress^25,26^. Warm waters affect the reproductive phenology, development and survival of marine larvae^27^. This warming may also increase the levels of local retention in populations, resulting from the faster larval development during the pre-competency period^28^. This thermal stress by warm and cold ENSO events is considered the main threat to ETP reefs^29,30^. Recently, extreme ENSO events in 1982–83 and 1997–98 have caused the localised collapse of many ETP coral reefs^25,31^. Ocean heatwaves produced by El Niño events are expected to increase in frequency and intensity, generating more thermal stress and mass bleaching events^31^.

Some evidence suggests that after massive disturbance events, the rescue of ETP pocilloporid populations is possible by recruitment of larvae originating in distant CTP populations^32^. Other studies suggest that rescue depends exclusively on self-recruitment^22^ and thermal refuges^33^. Most of the ETP coral reefs (except in the Western and Eastern Galapagos Islands^34^) have recovered their coral cover in the past two decades suggesting some level of resilience^35,36^. However, it is unclear whether coral recovery depends on larvae arriving from distant-source populations (CTP or regionally) or those being produced and retained locally^32^.

Geographic distances between coral reefs in the ETP exceed the average potential dispersal distances reported for other regions such as the Caribbean and Indo-Pacific (e.g., 50–200 km^37–39^), which suggests that long-distance larval dispersal may not play a major role in population dynamics in the ETP. The lack of direct techniques to track planktonic larvae makes measuring long-distance dispersal and quantifying connectivity patterns challenging. Although a diversity of indirect methods have been employed to quantify connectivity, including chemical marks, parentage analysis, population genetics approaches, and biophysical modeling^40^, recent advances in biophysical modelling have provided a robust framework to develop detailed and testable predictions^41^. This modelling approach combines ocean current data with seascape habitat maps and life-history traits such as reproductive strategy and larval characteristics to estimate the dispersal potential for marine organisms^42^.

Here, we used a spatially-explicit biophysical model of larval dispersal for the seascape including the Central and Eastern Tropical Pacific to develop biologically-realistic estimates of population connectivity. A dispersal simulation tracks a cloud of virtual larvae (e.g., a cohort of larvae spawned at a source reef) as it moves through the seascape, dependent on habitat data, dynamic oceanography, and the biological characteristics of the species of interest^43^. Modelling this cloud of larvae through to settlement allowed us to determine the strength and structure of the functional population connectivity across the entire region. These data were used to assess the long-distance dispersal hypotheses of key reef-building species crossing the EP-Barrier. We also evaluated whether the intensity of ENSO events is associated with the ability of coral larvae to breach the EP-Barrier. Lastly, we highlight the ecoregion-scale connectivity in the ETP and discuss its management implications.

## Results

### Central and Eastern Tropical Pacific seascape-wide connectivity

**Figure 1:**
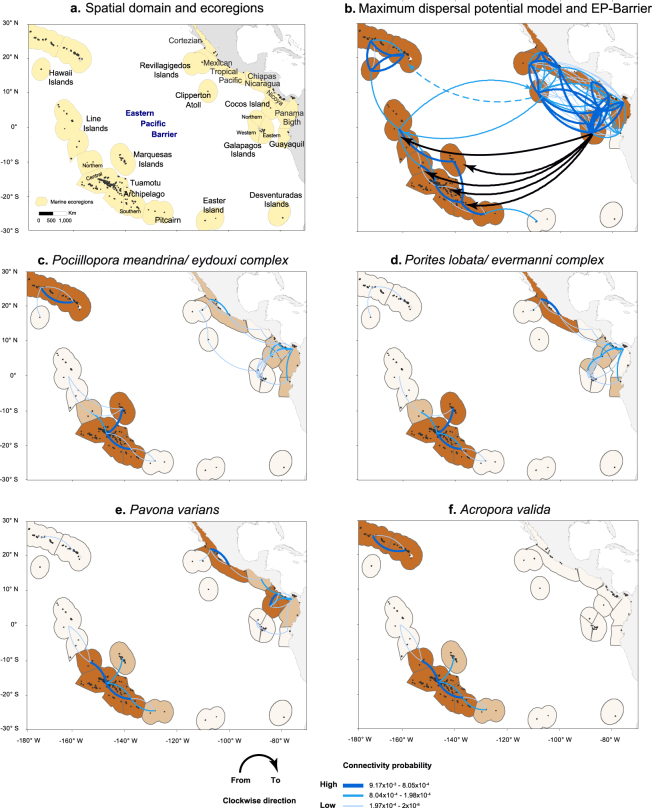
Spatial domain and coral connectivity networks across the EP-Barrier. (**a**) Ecoregions within the CTP and ETP. (**b**) The DPM_max_ connectivity network and main westward and eastward dispersal routes for breaching of the EP-Barrier. The dashed line indicates the route to Hawaii Islands. (**c**) *Pocillopora*_PLD150_ model with connection strength depicted in colour/weight from low connectivity strength (60% with dispersal probabilities between 1 × 10^−4^ and 1 × 10^−6^) to high (16% with dispersal probabilities greater than 0.01). (**d**) *Porites* model. (**e**) *Pavona varians*. (**f**) *A. valida*. Connectivity between ecoregions is represented by links above the migration rate threshold. Black areas represent the location of coral reef habitat. Ecoregions with high internal connectivity are shown in dark orange across the CTP and ETP. Maps were created with ArcGIS 10.3.1 using data sources described in the Methods.

We estimated the functional connectivity of the coral populations between the CTP and ETP. The analysis included 20 years of data from 1993 to 2012 of daily surface currents in 23 ecoregions (Fig. 1a). We developed individual models to evaluate six connectivity scenarios using key reef-building coral species with different larval competency characteristics and spawning phenologies. The models were: Maximum dispersal potential model (DPM_max_)*, Pocillopora*_PLD150_, *Pocillopora*_PLD100_, *Porites*_PLD50_, *P. varians*_PLD30_, and *A.valida*_PLD120_ (the parameters and outputs are detailed in Methods and Supplementary information). We found bi-directional connectivity across the EP-Barrier for DPM_max_ but did not find cross-ETP dispersal for any of the individual reef-building coral scenarios (above the migration rate threshold of 1 × 10^−6^, see Methods). We explain the major differences between the six connectivity models below.

#### DPM_max_

A virtual species with a 150-day pelagic larval duration (PLD), low larval mortality, and the ability to reproduce and spawn year-round was used. This resulted in an open population that received and exported individuals to most of the populations across the entire seascape. We found two main dispersal paths for breaching the EP-Barrier (Fig. 1b). The strongest and most frequent was a westward dispersal path from the Galapagos Islands in the ETP to the Marquesas Islands, Tuamotu Archipelago, and Line Islands in the CTP (Fig. 1b). The second strongest dispersal routes included both eastward and westward paths: eastward from the Line Islands in the CTP to Clipperton Atoll in the ETP, and westward from Clipperton Atoll to the Hawaii Islands in the CTP.

#### Pocillopora models

The connectivity patterns for the *Pocillopora*_PLD150_ and *Pocillopora*_PLD100_ models were similar, with neither showing dispersal capacity across the EP-Barrier. The highest connectivity probability was found within the Hawaiian Islands and between the Marquesas Islands and the Tuamotu Archipelago (Fig. 1c). In all of the scenarios explored, isolation was evident in remote ecoregions such as the Hawaiian Archipelago in the Northwest, and Easter Island and the Desventuradas Islands in the Southeast. In the Tuamotus, Rapa-Pitcairn, and Marquesas ecoregions, the primary surface currents flow southward, making Central and South Tuamotus local stepping-stones for dispersal. All of the ETP ecoregions were connected in both the *Pocillopora*_PLD150_ and *Pocillopora*_PLD100_ models; the strongest connections were in the southern ecoregions of Nicoya, Cocos Island, and the Panama Bight, which form a connectivity loop (for definition see Table S6). We recorded connections from Nicoya and Chiapas-Nicaragua to the Mexican Tropical Pacific ecoregion, potentially diminishing the Central American Faunal Gap (i.e., Chiapas-Nicaragua Ecoregion) as an oceanographic barrier for dispersal. The Cortezian and Revillagigedos ecoregions were connected to the continental Mexican Tropical Pacific but isolated from other south-eastern continental ecoregions.

#### Porites model

The highest connectivity was between the Marquesas Islands and the Tuamotu Archipelago. This model showed Clipperton Atoll being isolated from all other ecoregions (Fig. 1d). In the ETP, the Cortezian ecoregion was connected to the Mexican Tropical Pacific and Revillagigedos ecoregions but isolated from other southern ecoregions. The Mexican Tropical Pacific was connected only from Chiapas-Nicaragua and Nicoya, while Nicoya, the Panama Bight, and Cocos Island ecoregions formed a connectivity loop.

#### Pavona varians

The highest connectivity probabilities were found within the Tuamotu Archipelago. The population connectivity structure was similar to the *Porites* model (Fig. 1e). In the ETP, the connectivity loop linking the Cocos Island, Nicoya, and the Panama Bight ecoregions persisted; however, the link from Panama to the Galapagos Ecoregions was lost. For *P*. *varians*, the Galapagos ecoregions were only connected to the Guayaquil ecoregion, forming an isolated cluster.

#### Acropora valida

The populations showed high connectivity within the Tuamotu Archipelago and Hawaii Islands ecoregions; the Hawaii Islands, however, were isolated from all other ecoregions (Fig. 1f). It should be noted that *A. valida* is not currently found in the ETP.

### EP-Barrier routes and their association with ENSO events

#### Line Islands-Clipperton Atoll dispersal route (~5,000 km)

**Figure 2:**
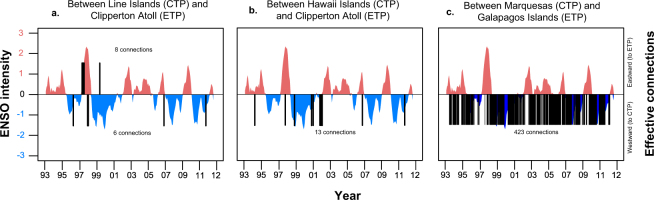
Significant connection strength for breaching the Eastern Pacific Barrier overlaid on ENSO events spanning 1993 to 2012. Out of 470 dispersal simulations, successful connectivity events across the EP-Barrier are shown for: (**a**) Between Line Islands (CTP) and Clipperton Atoll (ETP); (**b**) Between Hawaii (CTP) and Clipperton Atoll (ETP) and; (**c**) Between Marquesas (CTP) and Galapagos Islands (ETP). To illustrate the effect of positive and negative ENSO intensities in relationship to the direction of connectivity, effective connection events were plotted as black bars for eastward (above horizontal line) and westward (below) directions. The absence of a vertical bar implies there is no connection. Figure created with R 3.3.0 using data sources described in the Methods section.

**Figure 3:**
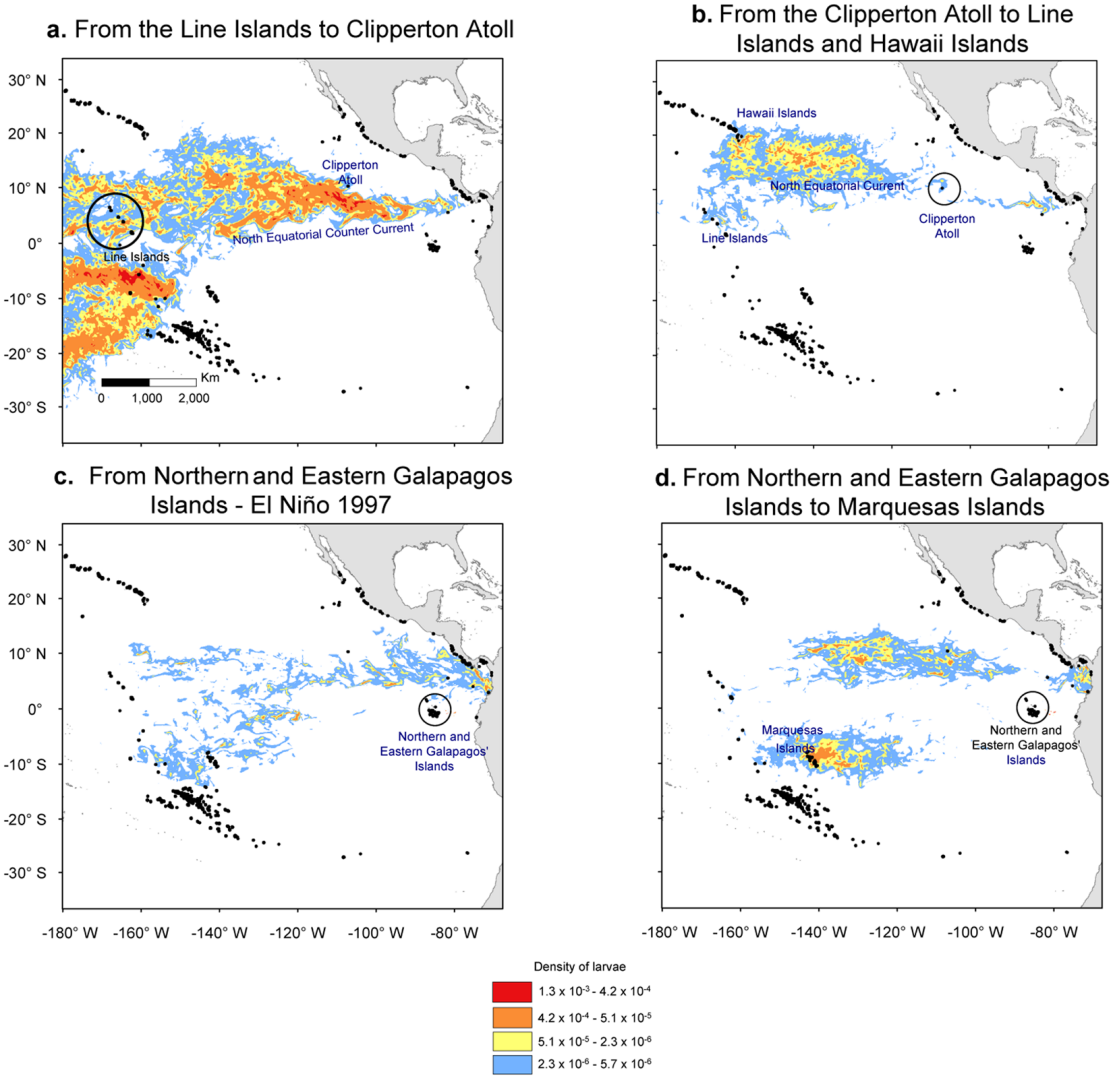
Larval density across the Eastern Pacific Barrier during the El Niño event of 1997–98. (**a**) From the Line Islands to the Clipperton Atoll the larvae dispersed following the NECC. (**b**) From the Clipperton Atoll to the Line and Hawaii Islands the larvae followed the NEC. (**c**) From the Galapagos Islands during El Niño 1997–98 when the NEC stopped its flow. (**d**) Continuous dispersal from the Galapagos’ Islands to the Marquesas Islands during multiple dispersal events 1993–2008. Larval densities were estimated for the DPM_max_ model and represent the additive densities of 20 simulations (b, c, and d) and five simulations (a) according to the likelihood of breaching the EP-Barrier (Fig. 2). Maps were created with ArcGIS 10.3.1.

We used the DPM_max_ simulations to test the dispersal route between the CTP and ETP. We found a bi-directional dispersal crossing through the EP-Barrier resulting from rare, long-distance dispersal events during extreme El Niño seasons in 1997–98 (Figs 2a,b and 3a,b). Between May and August 1997, seven out of 470 possible connections occurred eastward from the Line Islands to the Clipperton Atoll and one in June 1999 during weak La Niña conditions. Larval transport from the Line Islands to the Clipperton Atoll was 120–130 days. In the opposite direction, from the Clipperton Atoll to the Line Islands (Figs 2b and 3b), we observed six out of 470 possible connections with a larval transport time between 145–150 days. We also found a connection in May 1996 (neutral ENSO), three in January and February 1998 (extreme El Niño), another in January 2007 (weak El Niño), and one in January 2012 (weak La Niña). Although transpacific connections occurred mainly during El Niño events (e.g., 1997–98), breaching of the EP-Barrier was not strictly related to ENSO intensity (Table S5).

#### Clipperton Atoll-Hawaii Islands dispersal route (~5,100 km)

We observed 13 out of 470 possible connections with a larval transport time of approximately 140 days and a cumulative probability of connectivity of 7.0 × 10^−5^. Ten connections occurred during La Niña and neutral ENSO events, and three connections during El Niño events (Figs 2c and 3b). Connections during La Niña events occurred in January 1999, February 2001, and December and January 2012. Connections during neutral ENSO conditions occurred in April 1994, and January and February 2002. Connections during El Niño events occurred in December 1997 and December 2006. The strength of these connections was not related to ENSO intensity (Table S5). All the connections occurred when larvae were released during December, January, and February, except for one that resulted following a release in April.

#### Galapagos Islands-Marquesas Islands dispersal route (~4,900 km)

This route was the primary dispersal pathway crossing the EP-Barrier with connectivity probabilities ranging from 1.3 × 10^−2^ to 9.5 × 10^−3^. We observed 333 of 470 possible connections and 90 of 470 connections from the Eastern and Northern Galapagos Islands to the Marquesas Islands, respectively (Figs 2d and 3c,d). This westward dispersal route cross of the EP-Barrier may occur regularly, interrupted by moderate to high-intensity El Niño events in 1997, 2002, and 2010 but not strongly associated with ENSO intensity (Table S5). The highest dispersal probability occurred during a neutral ENSO event in March 2003 with a larval transport time of 105–110 days. The highest frequency of dispersal events occurred in July and December across all years assessed. The years with the highest incidence of dispersal events were 1995, 2007, and 2010. The eastward route across the EP-Barrier was not achieved with transport times less than 100 days; however, this transport time did maintain the connections between the Galapagos Islands and the CTP.

### Ecoregion-scale connectivity within the Eastern Tropical Pacific

**Figure 4:**
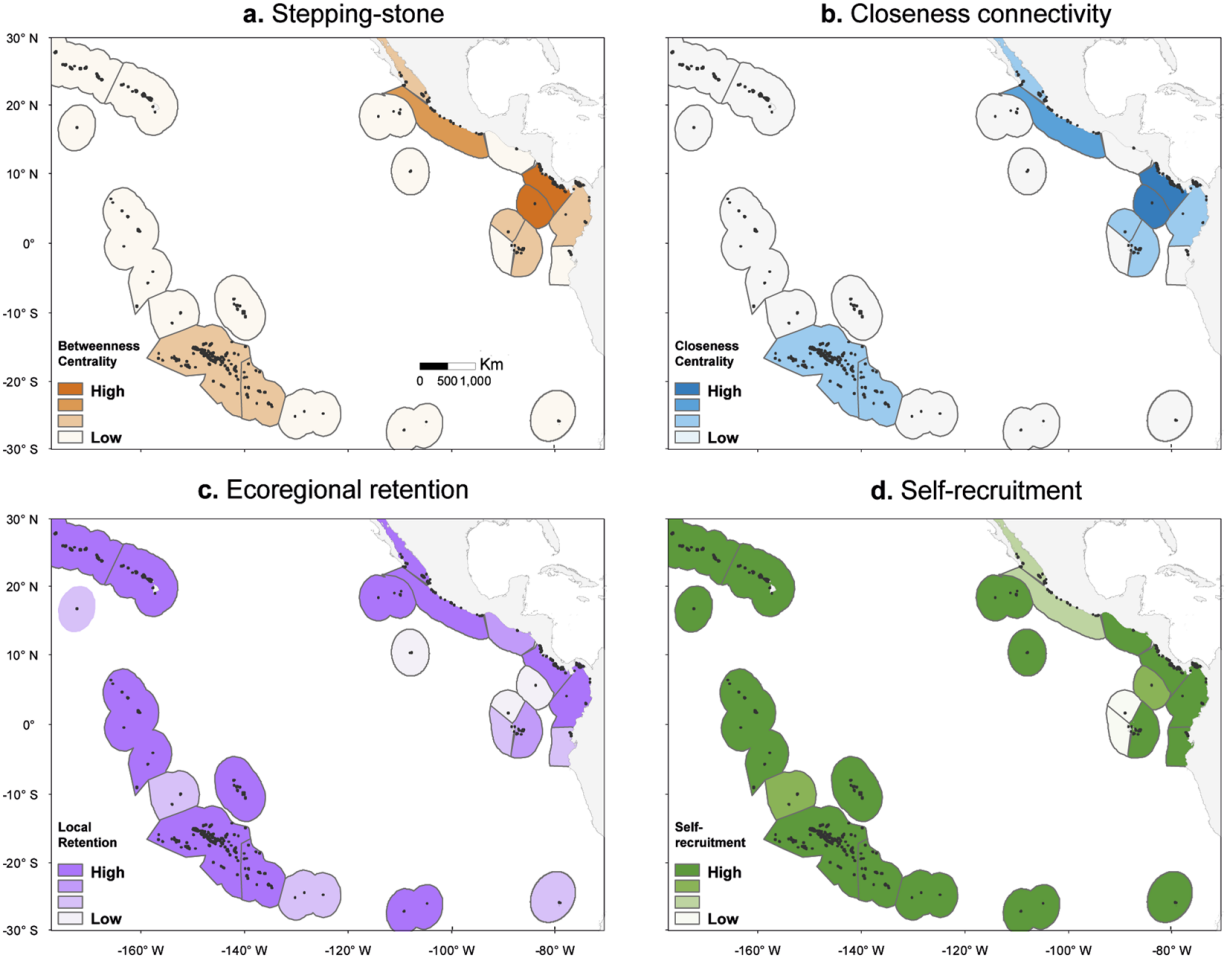
Ecoregion-scale connectivity. (**a**) The *Pocillopora*_PLD150_ model stepping stones scores. (**b**) Closeness centrality measures how close an ecoregion is to all other ecoregions in the network, (**c**) ecoregional-retention. (**d**) self-recruitment within ecoregions. Low to high values of each connectivity measure are indicated by colour intensity. Maps were created with ArcGIS 10.3.1.

Connectivity for *Pocillopora*, *Porites*, and *Pavona* in the ETP was characterised by northward flow along the coast from Nicoya and Chiapas-Nicaragua to the Mexican Tropical Pacific. We found a strong connectivity loops (believed to improve population persistence see definitions in Table S6) between the Nicoya, Panama Bight, and Cocos Island ecoregions, and within the Galapagos Islands (Fig. 1c–e) for these three reef-building coral species. In addition, we identified three key stepping-stones (high betweenness centrality, Table S7) ecoregions, including the Cocos Island, Nicoya, and the Mexican Tropical Pacific ecoregions (Fig. 4a).

Clipperton Atoll was a critical stepping-stone only in the DPM_max_ model. The Cortezian, Eastern Galapagos Islands, and Guayaquil ecoregions were most centrally located within the dispersal network revealed by high closeness centrality (Fig. 4b). Clipperton Atoll received dispersal connections from two sites, one from Northern Galapagos Islands, and one from Revillagigedos ecoregions, yet did not serve as a source of larvae for any other ecoregion (i.e., no outgoing connections). Revillagigedos also received one connection from the Cortezian ecoregion, and acted as a stepping-stone to Clipperton Atoll.

The ecoregional-retention or locally-produced larvae for the modelled species showed maximum levels in the range 0.63–0.83 in the Panama Bight, Nicoya, Cortezian, Mexican Tropical Pacific and Revillagigedos ecoregions (Fig. 4c,d). Clipperton Atoll, Cocos Island, Guayaquil, and Galapagos Islands ecoregions had extremely low levels of ecoregional-retention (0–0.20), implying that the vast majority of larvae were exported. Ecoregional self-recruitment for all species and ecoregions (except Northern and Western Galapagos Islands) was greater than 0.89, suggesting the dominance of locally-produced larvae in those eventually settling within ecoregions (Table S7). The ecoregion-scale connectivity metrics for the CTP can be found in Appendix 3.

## Discussion

### Seascape-wide connectivity

We explored the hypotheses of long-distance larval dispersal across the EP-Barrier and assessed connectivity strength and structure using a biophysical model of larval dispersal for five key reef-building species, as well as a virtual-species, a DPM_max_, represented by long larval durations, low larval mortality, and continuous spawning throughout the year.

As suggested by previous research^21,22^, most of the dispersal scenarios explored revealed that the CTP and ETP coral populations are not connected at this scale and dependent on local retention and larval recruitment from within the ecoregions. The virtual-species scenario, DPM_max_, was the sole exception. The resulting DPM_max_ network suggested that strong surface currents such as the North Equatorial Current (NEC) and South Equatorial Current (SEC), together with long larval durations could result in dispersal connections in both directions between the CTP and ETP, but primarily westward from Galapagos Islands to the Marquesas, Line, and Tuamotu ecoregions. The DPM_max_ model was designed using 2% mortality to differentiate the virtual larvae from inert particles such as pollutants, marine debris or buoyant plastic. The findings of our DPM_max_ model are comparable to those by Wood *et al*.^39^, which modelled dispersal using continuously released larvae over time. Overall, these studies agree that crossing the EP-Barrier occurred at various times under different ENSO states and in a predominately westward direction, which contradicts earlier biogeographic hypotheses suggesting eastward routes from the CTP^12,14^.

However, our DPM_max_ scenario differed in that we found strong support for bi-directional connectivity across the EP-Barrier. Perhaps, driven by species-specific biologic parameterisations such as the extended 150 d larval duration, daily mortality of 2%, and coral-specific spawning phenology (instead of the 120 d, 0.02 per day and no specific spawning phenology found by Wood *et al*.^39^). After establishing the importance of ETP coral spawning phenology in our previous work^26^, this current study extends the approach by Wood *et al*.^39^, and begins to address new questions related to ENSO influence on spawning phenology and connectivity in the region.

The biologic parameterisation is critical when modelling the biophysical processes of larval dispersal. We choose a maximum PLD of 150 and 100 d to simulate the key reef-building genus *Pocillopora*, given that the maximum for all broadcast-spawning scleractinian corals ranges from 195 to 244 d^44^ (see Appendix 2). However, the actual maximum PLD of ETP pocilloporids is unknown. Pocilloporid corals are distributed over thousands of kilometres suggesting historical or recent long-distance dispersal capabilities. Contrary to this observation, our *Pocillopora*_PLD150_ results yielded low connectivity strength and no connections between the CTP and ETP (Fig. 1c). Should eastward connections exist, they may occur as rafting or rare dispersal pulse events with larval durations exceeding 140 d and extremely low larval mortality (see next section). The *Pocillopora*_PLD150_ model results were consistent with other broad-scale biophysical connectivity models for benthic fauna, highlighting the influence of the EP-barrier in limiting eastward dispersal^21^, the isolation of the Hawaiian Archipelago^45^, and the isolation of ETP ecoregions from the Central Pacific^46^. The strength of the ETP dispersal connections was at levels 10 to 100-fold lower than those generated by studies in Micronesia^47^ and the Indo-West Pacific^48^.

The ecoregions of Easter Island and the Desventuradas Islands were isolated from other neighbouring ecoregions such as Rapa-Pitcairn, Tuamotus, and the Marquesas; this may be indicative of the presence of a strong southern dispersal barrier. Glynn *et al*. described this barrier previously^49^, suggesting that the existing distribution of pocilloporids in the Easter, Salas and Gomez and Desventuradas Islands could be the result of a range expansion during interglacial periods that used seamounts as potential stepping-stones.

### Crossing the Eastern Pacific Barrier

It has been hypothesised that pocilloporids cross the EP-Barrier from the CTP to the ETP using Clipperton Atoll as a stepping stone^32,50^. In the *Pocillopora*_PLD150_ model, we did not detect connections from the Line Islands to Clipperton Atoll or from Clipperton Atoll to any insular or continental ETP coral populations. In this model, Clipperton Atoll was a destination only for larvae from other ETP ecoregions. In the *Pocillopora* dispersal simulations, the larvae released from Clipperton Atoll did not reach continental habitats (Fig. 1c); most of the larvae travelled westward; a small portion moved eastward to the NEC, around 110°W, where they were advected westward.

Assuming that rare or pulse dispersal events are the main mechanism to breach the EP-Barrier^16^, two conditions must be met to achieve this crossing from the Line Islands eastward to Clipperton Atoll. First, the NECC’s eastward flow must increase during strong El Niño events^18^. Second, these events should stimulate the coral’s reproductive activity by triggering earlier or shorter gametogenesis and spawning in the Line Island and Clipperton Atoll.

Concerning the first condition, we found that during the extreme El Niño events in 1997–98^21^, the NECC increased its eastward surface flow. Larval transport time from the Line Islands to Clipperton Atoll was 110–130 days exceeding previous transport time estimates (e.g., 50–120 days^14,16–18^). However, during these 1997–98 events, westward breaching of the EP-Barrier from Clipperton Atoll to the Line Islands and the central Hawaiian Islands was also possible. Connections to the Line Islands were observed during strong (1998/01 and 1998/02) and neutral (2007/01 and 2012/01) ENSO conditions. Noticeably, the sporadic acceleration of the NECC is unclear.

On the second condition, it is suggested that ENSO’s positive temperature anomalies can influence the reproductive activity of pocilloporids^25^. In addition, pocilloporid oocyte maturation and spawning is likely to occur in the ETP in water temperatures ranging from 24–29 °C^26^. Water temperature from 1997/01 to 1998/12 (Appendix 1) in the Clipperton Atoll and the Line Islands, which includes the period during the strong 1997/98 El Niño events, did not exceed 30 °C. There were no reports of coral mortality in 1997/11^35^. Therefore, bi-directional transport may be more likely in warm (but not stressful) water temperatures that favour coral reproduction^26^. In pocilloporids, there is currently no evidence of a trade-off between higher water temperatures and increased reproductive activity and shorter developmental periods^28^. Pocilloporids, however, show sign of hosting the stress-tolerant *Symbiodinium glynni*, which may provide them with some resistance to bleaching^51^. It remains unclear whether this thermal resistance is transferred to their larvae, making them beneficiaries of the potential warm water and time of spawning trade-off and thereby enabling long-distance dispersal^52,53^. Research on the trade-offs between Pocilloporids, coral holobionts, and the warm environment remains an ongoing research focus.

Different to previous works^32,50^, our study identified the Northern Galapagos Islands as a critical stepping-stone connecting the CTP and ETP in addition to Clipperton Atoll. Westward larval dispersal from the Galapagos Islands to the Marquesas Islands may be a persistent process influenced by the constant flow of the SEC. The absence of dispersal connections in the *Pocillopora*_PLD150_ model was partially due to the low reproductive output resulting from the low coral cover (<10%) observed over the last decades in the Galapagos Islands^54^. This isolation may drive the evolutionarily significant divergence between CTP and ETP populations. Recently, Darwin Island in the Northern Galapagos Islands has recovered up to 30% of its coral cover^34^, suggesting that it may become a key stepping-stone to the CTP if this recovery continues and reproductive output increases.

The model of *A. valida* was driven by a single observation made 35 years ago, where three colonies of this coral were collected in the Gorgona Island after a strong El Niño event in 1982^55^. This acroporid coral is found in the Line Islands^56^ but not Clipperton Atoll^57^. It lacks maternally inherited zooxanthellae and has a maximum pelagic larval duration of 100–130 days^57^. Connectivity of *A. valida* in the ETP was very rare in our model, which would support the alternative assumption that historical populations may no longer persist. The mechanism(s) by which this species crossed the EP-Barrier is unclear^58^. Alternate hypotheses for long distance dispersal include polyp clustering^59^, pumice^60^, and debris^61^ rafting, as well as an eastward flow via the NECC and the Equatorial Subsurface Countercurrents^62^.

The spatially explicit hypotheses presented here could be used to evaluate gene flow or genetic differentiation data to build a better understanding of the processes driving population connectivity and genetic divergence across the CTP and ETP^43,63^. Although the present spatial resolution (i.e., at ecoregion-scales) may be inappropriate for a robust analysis exploring the correlation between our modelled connectivity estimates and those based on genetic data for pocilloporid populations^23,64^, broad-scale sampling of pocilloporid corals has shown a wide-ranging historical gene flow across the Tropical Pacific, suggesting the potential for transpacific dispersal in three *Pocillopora* species^24^. A recently published review^20^ further discusses patterns of connectivity using F_ST_ statistics for corals, gastropods, echinoderms, and fishes.

### Conservation considerations in the Eastern Tropical Pacific based on connectivity

For the first time, we described the formation of a connectivity loop between the Nicoya, Panama Bight, and Cocos Islands ecoregions, and within the Galapagos Islands. This connectivity loop is generated by cyclonic and anti-cyclonic gyres in the Panama Bight^65^, as well as by the seasonal influence of the NECC, whose eastward flow is strong across this region in the second part of the year^66^. Connectivity loops have been shown to be advantageous in promoting the persistence of metapopulations^67,68^.

The downstream connections along the coast from Nicoya to the Mexican Tropical Pacific in the ETP result from the Costa Rica Coastal Current (CRCC) and the West Mexican Current (WMC), respectively^66^. For the *Pocillopora* models, the Revillagigedos ecoregion is a key stepping-stone along a corridor running southward from the Cortezian ecoregion to the Clipperton Atoll likely explaining their strong coral fauna similarities^19^. Connections were not found from Revillagigedos to Clipperton Atoll in the *Porites* and *P. varians* models, however, *P. lobata* at the Clipperton Atoll was genetically similar to populations in the Central Pacific^22^.

Gyres reducing downstream larval transport produce semi-permeable barriers throughout the ETP. It is hypothesised that the region’s south-westward eddy activity, primarily at the entrance of the Gulf of California, acts as a barrier separating peninsular and continental populations^69^. Mesoscale eddies in the Gulf of Tehuantepec, Papagayo, Panama^70^, as well as the Tehuantepec Bowl and the Costa Rica Dome^66^ may trap or redirect larvae offshore or impede their northward dispersal along the American coastline. This eddy activity in the Gulf of California entrance, may also explain the weak connectivity between the Mexican Tropical Pacific and Cortezian ecoregions. Our results coincide with the north-westward gene flow direction reported for the populations of *Porites panamensis*^71^. In the Gulf of Tehuantepec, the CRCR flows south, feeding the Tehuantepec Bowl and also interrupting the westward flow to the WMC^66,72^. However, our simulations suggest that some branches of the CRCR flow north-westward, crossing the Gulf of Tehuantepec. Kessler^66^ proposed that during the summer, the Tehuantepec Bowl weakens and retreats offshore; this coincides with the spawning period for many coral species.

High values of self-recruitment (i.e., the proportion of total settlers to a site that originated in that site^43^) predominated in all of the ecoregions and modelled species (except the Galapagos Islands). These values suggest that these ecoregions are relatively closed to broad-scale immigration and that the majority of successfully settled larvae are produced locally. On the other hand, ecoregional-retention, which quantifies the segment of larvae produced by a particular ecoregion that settle within the same ecoregion, contains information on local persistence through replacement^68^ as well as the demographic independence of populations^73^. With higher resolution products, such as HYCOM, it could be important to assess whether Clipperton Atoll and Cocos Island have low ecoregional-retention and the majority of larvae produced in these ecoregions are exported and therefore reliant on larval subsidies from other ecoregions. However, exploring this further requires better habitat data and a hydrodynamic model with higher spatial and temporal resolution. In contrast, the Panama Bight and Nicoya ecoregions showed high ecoregional-retention, implying that a significant portion of locally produced larvae recruit into the same ecoregion (or into itself a few generations later through a connectivity loop), which makes these ecoregions more likely to be self-persisting.

Most of the ETP coral reefs have recovered during the past two decades^35,36,74^, suggesting, at least for pocilloporids, the ETP populations can persist with very low levels of connectivity between patches. Increased levels of ecoregional-retention and self-recruitment in corals suggest that fine-scale conservation actions (e.g., reducing local stressors that affect coral cover) could be more effective than broad-scale management strategies such as developing MPA networks^28^. Although bidirectional dispersal pathways may exist between the ETP and CTP at a frequency of about one per decade, this low frequency and weak strength in connections suggest management decisions should primarily be locally-based^43^.

### Modelling caveats

The results of biophysical modelling presented here have some important caveats. First, there is some uncertainty about the location and abundance of reef habitat in some regions. For example, research efforts along central American coastlines have continuously updated the distributional records for coral assemblages^19^. Future biophysical modelling in the region should include these new and updated reef cover maps, as significant gaps previously existed. Reef habitat and the local abundance of reproductive adults can affect the total reproductive output, or source strength, of modelled populations. In addition, due to the lack of data, all coral habitat attributes (e.g., quality, percent-cover) influencing larval settlement and post-settlement survival were considered identical. Differences in thermal stress, habitat quality or phenotype environment mismatch^75^ could be included in the model to improve the predictions of realised connectivity once data become available.

Second, several assumptions were required regarding biological attributes. Although we strived for biological realism in our parameter estimates, there is still some uncertainty around the reproduction and larval biology for most of the ETP corals assemblages. Perhaps the most urgent needs are more observations of spawning; survey data on adult abundance, densities, and the reproductive output; as well as controlled experiments to measure larval traits such as buoyancy and competency. Most coral larvae have low swimming abilities^76^; in our models, this larval characteristic was not included and assumed to have a non-significant effect on broad-scale connectivity outcomes. Also, further development of the mortality rate function could include spatial-temporal variations in survival caused by changes in temperature, salinity, nutrients, or predation. These are areas of ongoing research.

Third, we were constrained by the availability of regional and validated hydrodynamic data. The available spatial resolution of the HYCOM hydrodynamic model (~9 × 9 km) adequately resolves mesoscale eddies and strong sub-regional hydrodynamic structures such as upwelling, coastal currents, and fronts, all of which significantly influence patterns of dispersal and connectivity. However, this model cannot resolve fine-scale hydrodynamics such tidal flows, and shallow-water and near-shore (or boundary layer) dynamics. As a result, it is likely that local-retention may be underestimated as retention often increases as a function of coastal hydrographic model resolution. Underestimating local retention can overestimate downstream connectivity. However, we believe that the influence of this on our results and interpretation is minor. ETP coral assemblages tend to be more closed systems because of the geographic distances between ecoregions, and our model and results reflect this basic pattern. Although biophysical models have explicit challenges and assumptions, once the physics and biology have been largely validated, they can often help predict the spatial genetic variation of marine organisms with a larval dispersal stage^21,47,77^.

## Conclusion

The coral assemblages of the CTP and ETP have weak regional population connectivity and are relatively closed to immigration. This weak connectivity implies that replenishment by recruitment is primarily local, that larval contributions to distant populations may be limited, and that rare transpacific dispersal events may have negligible demographic effects for pocilloporids across the EP-Barrier. The permeability of the EP-Barrier is largely dependent on seasonal and decadal cycles (e.g., El Niño events) that may help facilitate long-distance dispersal and gene flow across this seascape, as suggested in recent pocilloporid genetic studies. The bi-directional crossing of the EP-Barrier seems possible for long-lived larvae (>140 days) between the Line Islands and Clipperton Atoll, with rare long-distance dispersal events most likely occurring during strong ENSO events. Insular ETP ecoregions were the source of larvae arriving into Clipperton Atoll, which can function as a stepping-stone to the Line and Hawaii Islands for pocilloporids. The westward route crossing of the EP-Barrier from the Northern and Eastern Galapagos Islands to the Marquesas Islands is potentially a persistent process promoted by the constant flow of the SEC – yet restricted during strong El Niño events. However, the decline in coral cover in the Galapagos Islands over the past decades and subsequent reduction of larval output, likely weakens this potential westward dispersal route. For most of the species modelled, we identified network properties in the ETP that positively influence population persistence such as stepping-stones and connectivity loops, like those observed at Cocos Island, Nicoya, Panama Bight, and the Mexican Tropical Pacific ecoregions. Conservation and management strategies developed for coral population persistence across this seascape may benefit from a local ecoregional-scale, rather than a seascape-wide focus due to the high local settlement and often limited immigration from external ecoregions.

## Methods

We used a spatially-explicit larval dispersal model to accomplish our three objectives of quantifying seascape-wide connectivity, estimating the influence of ENSO events on the crossing of the EP-Barrier, and identifying connectivity-based conservation priorities. This modelling approach included three main components: a spatial seascape of reefs and land, a hydrodynamic model, and the species’ reproductive and dispersal traits. Each component is explained in detail below.

### Spatial domain and hydrodynamic model

The spatial domain extended from 180°W to 69°W, and from 34°N to 37°S. Using ArcGIS 10.3.1 (http://desktop.arcgis.com), we combined data from the Millennium Coral Reef Mapping Project Version^78^ and regional reef habitat data from the published literature to build a reef habitat layer. To create land/sea boundaries we used the Global Self-consistent, Hierarchical, and High-resolution Shoreline (GSHHS) databases^79^. All spatial data were rescaled to a 9 × 9 km gridded reef map consistent with the hydrodynamic data resolution, which resulted in a gridded spatial domain that contains 935 rows by 1373 columns of which 1265 are habitat cells that represent the source/settlement locations. We grouped habitat patches across the domain into 23 ecoregions, 12 for the ETP and 11 for the CTP (Fig. 1a). Hydrodynamic data for current velocities were obtained from HYCOM + NCODA Global Reanalysis - HYCOM Consortium (https://hycom.org/dataserver/glb-reanalysis) and extracted for 1993 to 2012 for the top 30 m of the ocean.

### Dispersal Model

We used a spatially-explicit larval dispersal model^41,43^ and represented the asynchronous spawning phenology of key hermatypic corals species. The species-specific biological attributes to parameterise the model were obtained in four steps (see details in Appendix 1 and 2). First, for the CTP and Hawaii Islands, we used as a framework a previous study^26^ and conducted a systematic search to determine the spawning month of the corals *Pocillopora meandrina/eydouxi* complex (hereafter referred to as *Pocillopora* model), *P. lobata/evermanni* complex (hereafter referred to as *Porites* model), *Pavona varians*, and *Acropora valida* (Table S2). Second, we combined these spawning records, with the spawning phenology of the ETP^26^ and built a comprehensive reproductive phenology for the entire spatial domain (Table S3). If the month of spawning was unknown for an ecoregion, we assumed the spawning occurred when the water temperature was at its maximum^26^ (Fig. S1). Third, we combined the CTP and ETP biological attributes information such pre-competency period, mortality, and PLD (Appendix 2), creating the species-specific parameters for the spatial domain. Lastly, the larval productivity of each grid habitat cell was scaled by the amount of habitat assumed in that cell (Table S4).

We built six dispersal scenarios to explore the cross-EP-Barrier connectivity (Table 1). The first model consisted in deriving a maximum dispersal potential model with 150-day PLD and 2% daily larval mortality and consistent spawning times occurring every full and new moon (Fig. 1b). We built two scenarios for the *Pocillopora* model with a maximum PLD of 150 and 100 days (_PLD150_ and _PLD100_, Fig. 1b), and another scenario for the remaining species, *Porites* (_PLD50_, Fig. 1d), *P. varians* (_PLD30_, Fig. 1e), and *A. valida* (_PLD120_, Fig. 1f). All the species had a 10% daily larval mortality.

**Table 1:** Description of the biological parameters used for the six scenarios in the biophysical modelling. Definitions follow^41,43^.

Larval Biological Parameter	Description	DPM_max_	*P. meandrina/eydouxi* complex	*P. lobata/evermanni* complex	*P. varians*	*A. valida*
Spawning timing	Date of larval release during spawning	Monthly, every full and new moon	Two to seven spawning events per year during at full moon according to ecoregion and species (see Table S3)			
Pre-competency period	After fertilisation, larvae require hours to days to reach a competency stage, that is, capable of settlement and metamorphose	We applied the Gamma cumulative distribution function to represent the onset of larval settlement competency. We used the parameters 16 and 0.25 that imply a 50% competent larvae after 4 days				
Daily larval mortality	The daily mortality rate for a negative exponential decay of larvae while dispersing	2%	Larval mortality is unknown for the modelled species, though it is reported in the order of 5% to 10% day^−1^ (see details in^80^)			
Maximum pelagic larval duration (days)	The length (days) of the maximum larval dispersal period	150	150 and 100	50	30	120
Settlement Rate	Rate at which competent larvae will settle when over the reef	0.95				
Larval behaviour	Swimming and homing capabilities of larvae (active or passive)	Passive, no homing				
Migration rate threshold	Lower probability threshold below which no migration was inferred	1/1 000 000				
Diffusivity	Diffusivity constant in m^2^s^−1^. Describes the biological-physical repulsion between larvae	100				

Once the biophysical model was parameterised with the reproductive and dispersal traits, we released a cloud of virtual larvae from all the possible source reef cells. The larval cloud spread throughout the seascape dependent on the biophysical parameters and was diffused, transported, and concentrated through space and time. The biological parameters, current velocity, and turbulent diffusion controlled the overall dynamics of the larval cloud^43^.

### Model output, seascape-wide and regional connectivity

Each simulation produced two 3-dimensional matrices, representing dispersal likelihoods and larval densities. The elements of the dispersal matrix described the probability, at each time-step, that larvae released from ecoregion *i* survived and settled in ecoregion *j*. The density matrix showed the mass of larvae instantaneously released from all reefs at each summarisation step and represented the larvae that settled and remained in the water column^43^. To represent the cumulative probability of potential connectivity for each scenario, we calculated a single connectivity probability matrix (**P**) and a single migration matrix (**M**) from individual dispersal matrices (Appendix 2). We transformed matrix **M** to a biophysical distance matrix (**D**) using log (**M**^−1^)^80^. In matrix **D**, one unit of biophysical distance is equivalent to a 10-fold decrease in the proportion of immigrant settlers^47^.

We applied 1 × 10^−6^ larvae as the migration rate threshold (MRT) or the probability above which demographical connectivity was inferred^43^; for example, 1 recruit out of a million larvae released. All the entries of **P** less than 1 × 10^−6^ were considered non-demographically significant and potentially having evolutionary significance. We used matrices **P** and **D** to estimate five connectivity metrics: degree, betweenness centrality, closeness centrality, self-recruitment, and local-retention^80^ (Table S7).

### Eastern Pacific Barrier

To determine whether changes in ENSO intensity affected the probability of coral larval dispersal between the CTP and ETP, we simulated 470 spawning events from all source reefs through time. In all the simulations the spawning occurred at new and full moons, with a maximum PLD of 150 days, 2% daily mortality, and no homing behaviour (i.e., DPM_max_). From each connectivity probability matrix, P, we created a vector containing indices of each nonzero element describing the bi-directional probabilities through time from/to the Line Islands - Clipperton Atoll, from Northern and Eastern Galapagos Islands to the Marquesas Islands, and from Clipperton Atoll to Hawaii Islands. Using a linear regression model, these dispersal probability indices (response variable) were regressed against corresponding monthly ENSO-3 region intensities; these were obtained from the NOAA Climate Prediction Center’s Extended Reconstructed Sea Surface Temperature (ERSSTv4) dataset (http://www.cpc.ncep.noaa.gov/data/indices/). As previously done, we used a connectivity threshold of 1 × 10^−6^.

### Data availability

Data generated or analysed during this study are included in the Supplementary Information files.

## Electronic supplementary material

Supplementary Information
