# Supplementary material for: The Eastern Tropical Pacific coral population connectivity and the role of the Eastern Pacific Barrier
Source: Sci Rep. 2018 Jun 19;8:9354. doi: 10.1038/s41598-018-27644-2 (PMC6008413; doi:10.1038/s41598-018-27644-2)
Supplement: Supplementary file 1 — Supplementary Information [file 41598_2018_27644_MOESM1_ESM.docx]

Electronic Supplementary Information

for

**The Eastern Tropical Pacific coral population connectivity and the**

**role of the Eastern Pacific Barrier**

Mauricio Romero-Torres^1^, Eric A. Treml^2^, Alberto Acosta^1^, David A. Paz-García ^3-4^

^1^Unidad de Ecología y Sistemática (UNESIS), Departamento de Biología, Pontificia Universidad Javeriana, Carrera 7 No. 40 – 62, Bogotá, Colombia.

^2^School of BioSciences, University of Melbourne, Parkville, Victoria 3010, Australia

^3^Marine Speciation and Molecular Evolution Laboratory. Department of Biological Sciences, Louisiana State University, Baton Rouge, LA 70803, USA.

^4^Laboratorio de Necton y Ecología de Arrecifes, Centro de Investigaciones Biológicas del Noroeste (CIBNOR), Calle IPN 195, Col. Playa Palo de Santa Rita Sur, 23096 La Paz, B.C.S., México.

**Contents**

**Appendix 1.** Methods to construct the reproductive phenology of reef-building species in the Central Tropical Pacific.

**Appendix 2.** Model parameters and model output.

**Appendix 3.** Regression analysis, connectivity metrics and network analysis for the Maximum dispersal potential model and reef-building species scenarios.

**Appendix 1**

**Methods to construct the reproductive phenology of reef-building species**

**in the Central Tropical Pacific**

**Sea Surface Temperature (SST) calculations.** SST data were obtained from the Physical Oceanography Distributed Active Archive Center (PO.DAAC v7.3) at the NASA Jet Propulsion Laboratory, Pasadena, CA (https://podaac.jpl.nasa.gov/dataset/JPL-L4UHfnd-GLOB-MUR). For each location we downloaded daily SST products using GHRSST Level 4 MUR Global Foundation Sea Surface Temperature Analysis with a 0.011deg/1.2km resolution for the period 2002/06 to 2013-04 (Table S1). Data were transformed to Celsius degrees. For each location, long-term mean SST was calculated by averaging monthly mean SST over the 12-year time series.

To establish the SST at the Clipperton Atoll (10.32 N, 109.25 W) and the Line Islands (Kiritimani 2.11 N, 157.5 W) spanning from 1997/01 to 1998/12, we used the Physical Oceanography Distributed Active Archive Center (PO.DAAC v8.4rc5). We selected the data set GHRSST Level 4 AVHRR_OI Global Blended Sea Surface Temperature Analysis (GDS version 2) from NCEI.

**Supplementary Table S1.** Localities and geographic information for Sea Surface Temperature data.

| **Localities** | **Geographical coordinates**  **at PO.DAAC** |
| --- | --- |
| **Thermally stable regime Central Pacific - Line Islands** | |
| Palmira Atoll | 6.21 N, 162.17 W |
| Kingman | 6.33 N, 162.16 W |
| Christmas Islands | 2.01 N, 157.58 W |
| Kiritimani | 2.11 N, 157.5 W |
| **Seasonally regime at Southern hemisphere** | |
| Tuamotu Archipelago | 16.0 S, 142.3 W |
| Pitcairn Islands | 24.3 S, 128.33 W |
| Marquesas Islands (Hiva Oa) | 9.6 S, 139.02 W |
| Southeast Polynesia (Scilly, Raiatea, Moorea, Tahiri)) | 17.47 S, 149.47 W |
| Easter islands, Chile | 27.0 S, 109.41 W |
| **Seasonally regime at Northern hemisphere** | |
| Hawaii (S) | 18.94 N, 155.56 W |
| Hawaii (N) | 22.27 N, 159.58 W |
| Johnston atoll | 16.75 N, 169.53 W |

**Comprehensive reproductive phenology.** Following Romero-Torres *et al*.^1^, for the Central Tropical Pacific and Hawaii Islands, we conducted a systematic search to determine the spawning month of the hermatypic corals of the genus *Pocillopora* spp*., Porites* spp*., Pavona* spp*.*and *Acropora* spp*.* We selected studies that contain inferred or direct field observations of the spawning. We selected tables, graphs and research statements that describe the spawning, the reproductive cycle or the reproductive condition of the species. We registered the presence/absence of spawning when the proportion of colonies or polyps with oocytes at stage IV were greater than 25%. If there were more than one observation per month during the same year, we considered the data as one observation. The database of observations of spawning per ecoregion is presented in Table S2. We defined as a response variable the number of observations of spawning (direct and indirect) per specie per month.

**Supplementary Table S2.** Reproductive attributes, months with mature oocytes (stage IV) of the dominant coral species of Central Tropical Pacific and Hawaii Islands.

| **Ecoregion** | **Country** | **Place/Reef** | **Specie** | **Reproductive mode** | **Sexual system** | **Methodology** | **Year of sampling** | **Months with ova stage IV or spawning** | **Reference** |
| --- | --- | --- | --- | --- | --- | --- | --- | --- | --- |
| Line Islands | United States of America | Palmira Atoll | *Acropora clathrata* | Broadcast spawner | Hermaphrodite | Observation laboratory | 2002 | Mar | ^2^ |
| Line Islands | United States of America | Palmira Atoll | *Acropora cytherea* | Broadcast spawner | Hermaphrodite | Observation laboratory | 2002 | Mar | ^2^ |
| Line Islands | United States of America | Palmira Atoll | *Acropora elseyie* | Broadcast spawner | Hermaphrodite | Observation laboratory | 2002 | Mar | ^2^ |
| Line Islands | United States of America | Palmira Atoll | *Acropora gemmifera* | Broadcast spawner | Hermaphrodite | Observation laboratory | 2002 | Mar | ^2^ |
| Line Islands | United States of America | Palmira Atoll | *Acropora humilis* | Broadcast spawner | Hermaphrodite | Observation laboratory | 2002 | Mar | ^2^ |
| Line Islands | United States of America | Palmira Atoll | *Acropora hyacinthus* | Broadcast spawner | Hermaphrodite | Observation laboratory | 2002 | Mar | ^2^ |
| Line Islands | United States of America | Palmira Atoll | *Acropora nobilis* | Broadcast spawner | Hermaphrodite | Observation laboratory | 2002 | Mar | ^2^ |
| Line Islands | United States of America | Palmira Atoll | *Acropora samoensis* | Broadcast spawner | Hermaphrodite | Observation laboratory | 2002 | Mar | ^2^ |
| Line Islands | United States of America | Palmira Atoll | *Acropora tenuis* | Broadcast spawner | Hermaphrodite | Observation laboratory | 2002 | Mar | ^2^ |
| Line Islands | United States of America | Palmira Atoll | *Acropora valida* | Broadcast spawner | Hermaphrodite | Observation laboratory | 2002 | Mar | ^2^ |
| Line Islands | United States of America | Palmira Atoll | *Acropora verweyi* | Broadcast spawner | Hermaphrodite | Observation laboratory | 2002 | Mar | ^2^ |
| Line Islands | United States of America | Palmira Atoll | *Acropora A* | Broadcast spawner | Hermaphrodite | Observation laboratory | 2002 | Mar | ^2^ |
| Line Islands | United States of America | Palmira Atoll | *Acropora cerealise* | Broadcast spawner | Hermaphrodite | Observation laboratory | 2004 | Mar | ^2^ |
| Line Islands | United States of America | Palmira Atoll | *Acropora cytherea* | Broadcast spawner | Hermaphrodite | Observation laboratory | 2004 | Mar | ^2^ |
| Line Islands | United States of America | Palmira Atoll | *Acropora humilis* | Broadcast spawner | Hermaphrodite | Observation laboratory | 2004 | Mar | ^2^ |
| Line Islands | United States of America | Palmira Atoll | *Acropora hyacinthus* | Broadcast spawner | Hermaphrodite | Observation laboratory | 2004 | Mar | ^2^ |
| Line Islands | United States of America | Palmira Atoll | *Acropora nobilis* | Broadcast spawner | Hermaphrodite | Observation laboratory | 2004 | Mar | ^2^ |
| Line Islands | United States of America | Palmira Atoll | *Acropora polystoma* | Broadcast spawner | Hermaphrodite | Observation laboratory | 2004 | Mar | ^2^ |
| Line Islands | United States of America | Palmira Atoll | *Acropora verweyi* | Broadcast spawner | Hermaphrodite | Observation laboratory | 2004 | Mar | ^2^ |
| Line Islands | United States of America | Palmira Atoll | *Acropora B* | Broadcast spawner | Hermaphrodite | Observation laboratory | 2004 | Mar | ^2^ |
| Line Islands | United States of America | Kingman Atoll | *Acropora cerealis* | Broadcast spawner | Hermaphrodite | Observation laboratory | 2002 | Mar | ^2^ |
| Line Islands | United States of America | Kingman Atoll | *Acropora cytherea* | Broadcast spawner | Hermaphrodite | Observation laboratory | 2002 | Mar | ^2^ |
| Line Islands | United States of America | Kingman Atoll | *Acropora humilis* | Broadcast spawner | Hermaphrodite | Observation laboratory | 2002 | Mar | ^2^ |
| Line Islands | United States of America | Kingman Atoll | *Acropora hyacinthus* | Broadcast spawner | Hermaphrodite | Observation laboratory | 2002 | Mar | ^2^ |
| Line Islands | United States of America | Kingman Atoll | *Acropora valida* | Broadcast spawner | Hermaphrodite | Observation laboratory | 2002 | Mar | ^2^ |
| Line Islands | United States of America | Kingman Atoll | *Acropora cytherea* | Broadcast spawner | Hermaphrodite | Observation laboratory | 2004 | Apr | ^2^ |
| Line Islands | United States of America | Kingman Atoll | *Acropora humilis* | Broadcast spawner | Hermaphrodite | Observation laboratory | 2004 | Apr | ^2^ |
| Line Islands | United States of America | Kingman Atoll | *Acropora hyacinthus* | Broadcast spawner | Hermaphrodite | Observation laboratory | 2004 | Apr | ^2^ |
| Line Islands | United States of America | Kingman Atoll | *Acropora nobilis* | Broadcast spawner | Hermaphrodite | Observation laboratory | 2004 | Apr | ^2^ |
| Line Islands | United States of America | Kingman Atoll | *Acropora tenuis* | Broadcast spawner | Hermaphrodite | Observation laboratory | 2004 | Apr | ^2^ |
| Southeast Polynesia | France | Society Archipelago, French  Polynesia | *Acropora acuminata* | Broadcast spawner | Hermaphrodite | Observation laboratory and field | 2002 | Sep | ^3^ |
| Southeast Polynesia | France | Society Archipelago, French  Polynesia | *Acropora austera* | Broadcast spawner | Hermaphrodite | Observation laboratory and field | 2002 | Nov | ^3^ |
| Southeast Polynesia | France | Society Archipelago, French  Polynesia | *Acropora cytherea* | Broadcast spawner | Hermaphrodite | Observation laboratory and field | 2002 | Oct | ^3^ |
| Southeast Polynesia | France | Society Archipelago, French  Polynesia | *Acropora globiceps* | Broadcast spawner | Hermaphrodite | Observation laboratory and field | 2002 | Oct | ^3^ |
| Southeast Polynesia | France | Society Archipelago, French  Polynesia | *Acropora hyacinthus* | Broadcast spawner | Hermaphrodite | Observation laboratory and field | 2002 | Oct | ^3^ |
| Southeast Polynesia | France | Society Archipelago, French  Polynesia | *Acropora lutkeni* | Broadcast spawner | Hermaphrodite | Observation laboratory and field | 2002 | Oct | ^3^ |
| Southeast Polynesia | France | Society Archipelago, French  Polynesia | *Acropora polystoma* | Broadcast spawner | Hermaphrodite | Observation laboratory and field | 2002 | Oct | ^3^ |
| Southeast Polynesia | France | Society Archipelago, French  Polynesia | *Acropora pulchra* | Broadcast spawner | Hermaphrodite | Observation laboratory and field | 2002 | Sep | ^3^ |
| Southeast Polynesia | France | Society Archipelago, French  Polynesia | *Acropora retusa* | Broadcast spawner | Hermaphrodite | Observation laboratory and field | 2002 | Sep | ^3^ |
| Southeast Polynesia | France | Society Archipelago, French  Polynesia | *Acropora retusa* | Broadcast spawner | Hermaphrodite | Observation laboratory and field | 2002 | Oct | ^3^ |
| Southeast Polynesia | France | Society Archipelago, French  Polynesia | *Acropora secale* | Broadcast spawner | Hermaphrodite | Observation laboratory and field | 2002 | Sep | ^3^ |
| Southeast Polynesia | France | Society Archipelago, French  Polynesia | *Acropora secale* | Broadcast spawner | Hermaphrodite | Observation laboratory and field | 2002 | Oct | ^3^ |
| Southeast Polynesia | France | Society Archipelago, French  Polynesia | *Acropora striata* | Broadcast spawner | Hermaphrodite | Observation laboratory and field | 2002 | Oct | ^3^ |
| Southeast Polynesia | France | Society Archipelago, French  Polynesia | *Acropora* cf. *divaricata* | Broadcast spawner | Hermaphrodite | Observation laboratory and field | 2002 | Oct | ^3^ |
| Hawaii | United States of America | Oahu | *Pocillopora damicornis* | Brooder |  | Observation laboratory, Ex-situ, aquaria | 1972-1976 | Apr | ^4^ |
| Hawaii | United States of America | Oahu | *Pocillopora damicornis* | Brooder |  | Observation laboratory, Ex-situ, aquaria | 1972-1977 | May | ^4^ |
| Hawaii | United States of America | Oahu | *Pocillopora damicornis* | Brooder |  | Observation laboratory, Ex-situ, aquaria | 1972-1978 | Jun | ^4^ |
| Hawaii | United States of America | Oahu | *Porites lobata* | Broadcast spawner |  | Observation laboratory, Ex-situ, aquaria | 1972-1979 | Jul | ^4^ |
| Hawaii | United States of America | Oahu | *Porites lobata* | Broadcast spawner |  | Observation laboratory, Ex-situ, aquaria | 1972-1980 | Aug | ^4^ |
| Hawaii | United States of America | Oahu | *Porites Compresa* | Broadcast spawner |  | Observation laboratory, Ex-situ, aquaria | 1972-1981 | Jun | ^4^ |
| Hawaii | United States of America | Oahu | *Porites Compresa* | Broadcast spawner |  | Observation laboratory, Ex-situ, aquaria | 1972-1982 | Jul | ^4^ |
| Hawaii | United States of America | Oahu | *Porites Compresa* | Broadcast spawner |  | Observation laboratory, Ex-situ, aquaria | 1972-1983 | Aug | ^4^ |
| Hawaii | United States of America | Enewetak, Marshall Islands and Kaneohe Bay | *Pocillopora damicornis* | Brooder |  | Ex-situ, aquaria | 1981 | Jun | ^5^ |
| Hawaii | United States of America | Enewetak, Marshall Islands and Kaneohe Bay | *Pocillopora damicornis* | Brooder |  | Ex-situ, aquaria | 1981 | Jul | ^5^ |
| Hawaii | United States of America | Enewetak, Marshall Islands and Kaneohe Bay | *Pocillopora damicornis* | Brooder |  | Ex-situ, aquaria | 1981 | Aug | ^5^ |
| Hawaii | United States of America | Hawaii (Kane'ohe Bay) | *Porites lobata* | Broadcast spawner | Gonochoric | Observation laboratory | 1997 | Jun | ^6^ |
| Hawaii | United States of America | Hawaii (Kane'ohe Bay) | *Porites lobata* | Broadcast spawner | Gonochoric | Observation laboratory | 1997 | Jul | ^6^ |
| Hawaii | United States of America | Hawaii (Kane'ohe Bay) | *Porites compresa* | Broadcast spawner | Gonochoric | Observation laboratory | 1997 | Jun | ^6^ |
| Hawaii | United States of America | Hawaii (Kane'ohe Bay) | *Porites compresa* | Broadcast spawner | Gonochoric | Ex-situ, aquaria, observation laboratory | 1997 | Jul | ^6^ |
| Hawaii | United States of America | Hawaii (Kane'ohe Bay) | *Montipora verrucosa* | Broadcast spawner |  | Observation laboratory | 1997 | Jun | ^7^ |
| Hawaii | United States of America | Hawaii (Kane'ohe Bay) | *Montipora verrucosa* | Broadcast spawner |  | Observation laboratory | 1997 | Jul | ^7^ |
| Hawaii | United States of America | Hawaii (Kane'ohe Bay) | *Montipora verrucosa* | Broadcast spawner |  | Observation laboratory | 1997 | Aug | ^7^ |
| Hawaii | United States of America | Hawaii (Kane'ohe Bay) | *Porites lobata* | Broadcast spawner |  | Observation laboratory | 1997 | Jun | ^7^ |
| Hawaii | United States of America | Hawaii (Kane'ohe Bay) | *Porites lobata* | Broadcast spawner |  | Observation laboratory | 1997 | Jul | ^7^ |
| Hawaii | United States of America | Hawaii (Kane'ohe Bay) | *Porites lobata* | Broadcast spawner |  | Observation laboratory | 1997 | Aug | ^7^ |
| Hawaii | United States of America | Hawaii (Kane'ohe Bay) | *Pavona varians* | Broadcast spawner | Gonochoric | Observation laboratory | 1997 | Jun | ^8^ |
| Hawaii | United States of America | Hawaii (Kane'ohe Bay) | *Montipora verrucosa* | Broadcast spawner | Hermaphrodite | Observation laboratory | 1997 | Jul | ^8^ |
| Hawaii | United States of America | Hawaii (Kane'ohe Bay) | *Montipora verrucosa* | Broadcast spawner | Hermaphrodite | Observation laboratory | 1997 | Aug | ^8^ |
| Hawaii | United States of America | Hawaii (Kane'ohe Bay) | *Montipora studeri* | Broadcast spawner | Hermaphrodite | Observation laboratory | 1997 | Jul | ^8^ |
| Hawaii | United States of America | Hawaii (Kane'ohe Bay) | *Montipora studeri* | Broadcast spawner | Hermaphrodite | Observation laboratory | 1997 | Aug | ^8^ |
| Hawaii | United States of America | Hawaii (Kane'ohe Bay) | *Montipora studeri* | Broadcast spawner | Hermaphrodite | Observation laboratory | 1997 | Aug | ^8^ |
| Hawaii | United States of America | Hawaii (Kane'ohe Bay) | *Porites lobata* | Broadcast spawner | Gonochoric | Observation laboratory | 1997 | Jun | ^8^ |
| Hawaii | United States of America | Hawaii (Kane'ohe Bay) | *Porites lobata* | Broadcast spawner | Gonochoric | Observation laboratory | 1997 | Jul | ^8^ |
| Hawaii | United States of America | Hawaii (Kane'ohe Bay) | *Porites lobata* | Broadcast spawner | Gonochoric | Observation laboratory | 1997 | Aug | ^8^ |
| Hawaii | United States of America | Hawaii (Molokini Islet) | *Pocillopora meandrina* | Broadcast spawner | Hermaphrodite | Observation laboratory | 1991 | Apr | ^9^ |
| Hawaii | United States of America | Hawaii (Molokini Islet) | *Pocillopora meandrina* | Broadcast spawner | Hermaphrodite | Observation laboratory | 1991 | May | ^9^ |
| Hawaii | United States of America | Hawaii (Molokini Islet) | *Pocillopora meandrina* | Broadcast spawner | Hermaphrodite | Observation laboratory | 1995 | May | ^9^ |
| Hawaii | United States of America | Hawaii (Molokini Islet) | *Pocillopora meandrina* | Broadcast spawner | Hermaphrodite | Observation laboratory | 1996 | May | ^9^ |
| Hawaii | United States of America | Hawaii (Molokini Islet) | *Pocillopora meandrina* | Broadcast spawner | Hermaphrodite | Observation laboratory | 1997 | Apr | ^9^ |
| Hawaii | United States of America | Hawaii (Molokini Islet) | *Pocillopora meandrina* | Broadcast spawner | Hermaphrodite | Observation laboratory | 1998 | May | ^9^ |
| Hawaii | United States of America | French Frigate Shoals | *Acropora*  *valida* | Broadcast spawner | Hermaphrodite | Observation laboratory | 1989 | Jul | ^10^ |
| Hawaii | United States of America | French Frigate Shoals | *Acropora*  *humilis* | Broadcast spawner | Hermaphrodite | Observation laboratory | 1989 | Jun | ^10^ |

**Supplementary Figure S1.** The observed SST to predict the coral spawning (when the water temperature is at its maximum). Tuamotu Archipelago (a), Marquesas Islands (b), Pitcairn Islands (c) and Easter Island (d). See Sea Surface Temperature calculations for methodology.


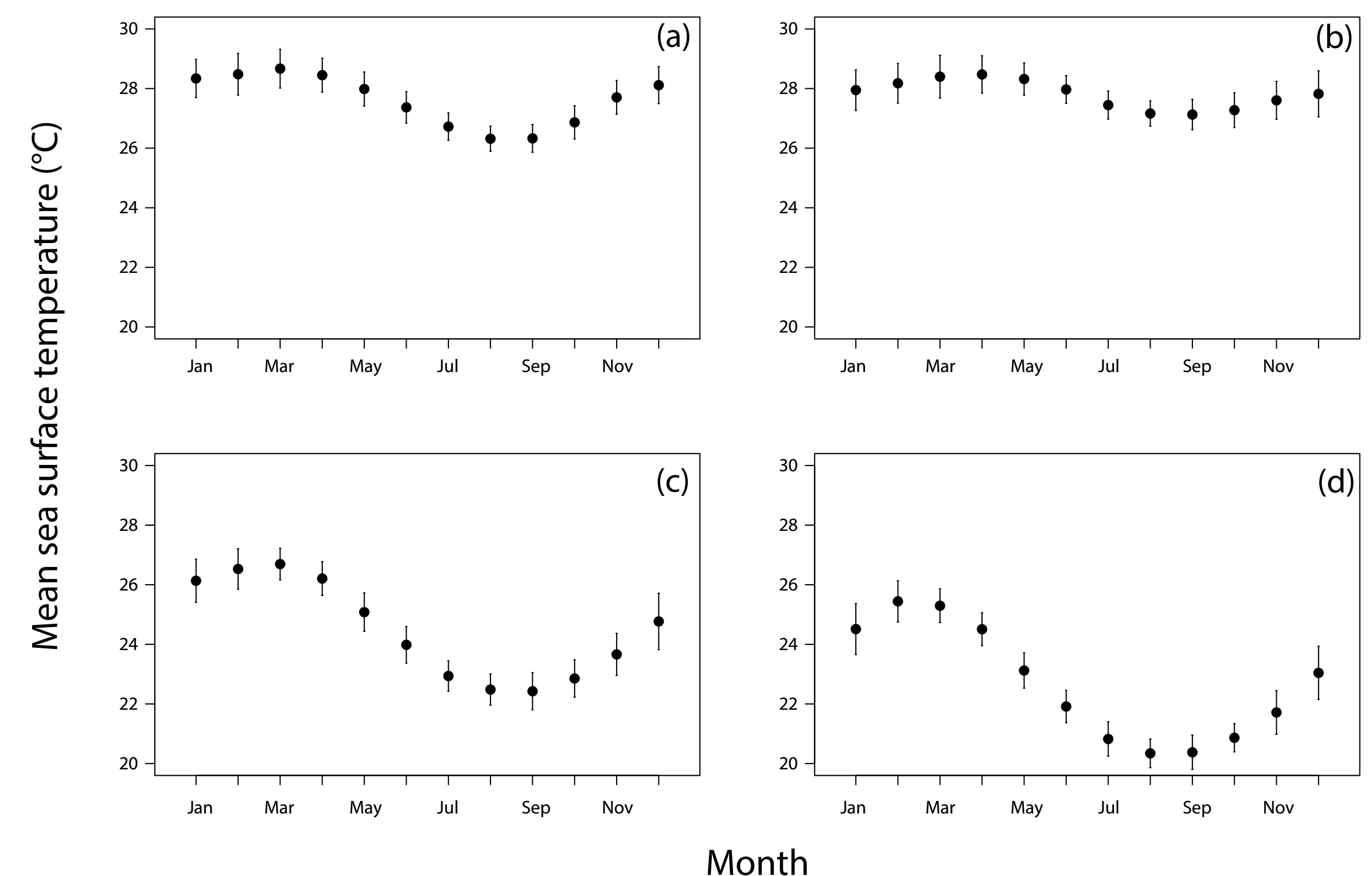


**Supplementary Table S3.** Spawing phenology and maximum pelagic larval duration used in the dispersal model across the eastern Pacific barrier. Numbers in species columns corresponds to spawning months observed or presumed (e.g. Fig. S1).

| **Region ID** | **Ecoregion** | ***P. meandrina/eydouxi complex***  **(PLD 150 and 100)** | ***P. lobata/evermanni complex***  **(PLD 50)** | ***P. varians***  **(PLD 30)** | ***A. valida***  **(PLD 120)** |
| --- | --- | --- | --- | --- | --- |
| 1 | Hawaii | 6,7,8 | 6,7,8 | 6,7,8 presumably | 6,7 |
| 2 | Hawaii | 6,7,8 | 6,7,8 | 6,7,8 presumably | 6,7 |
| 3 | Hawaii | 6,7,8 | 6,7,8 | 6,7,8 presumably | 6,7 |
| 4 | Line Islands | 7, 9,10,11 presumably | 7, 9,10,11 presumably | 7, 9,10,11 presumably | 3 |
| 5 | Tuamotus | 1,2,3 presumably | 1,2,3 presumably | 1,2,3 presumably | 9,10 |
| 6 | Tuamotus (Society Archipelago) | 1,2,3 presumably | 1,2,3 presumably | 1,2,3 presumably | 9,10 |
| 7 | Tuamotus | 1,2,3 presumably | 1,2,3 presumably | 1,2,3 presumably | 9,10 |
| 8 | Rapa-Pitcairn | 2,3,4 presumably | 2,3,4 presumably | 2,3,4 presumably | 9,10 |
| 9 | Marquesas | 2,3,4 presumably | 2,3,4 presumably | 2,3,4 presumably | 9,10 |
| 10 | Easter Island | 3,4 presumably | 3,4 presumably | 3,4 presumably |  |
| 11 | Desventuradas Islands | 3,4 presumably | 3,4 presumably | 3,4 presumably |  |
| 12 | Cortezian (GoC) | 8,9,10,11 | 8,9 presumably | 7,8,9,10 presumably |  |
| 13 | Mexican Tropical Pacific | 3,5,6,8,9,10,12 | 2,3,5,9,10 | 1,2,3,4,5 presumably |  |
| 14 | Revillagigedos | 8,9,10,11 | 8,9, presumably | 7,8,9,10 presumably |  |
| 15 | Clipperton | 3,5,6,8,9,10,12 | 2,3,5,9,10 | 1,2,3,4,5 |  |
| 16 | Chiapas-Nicaragua | 3,5,6,8,9,10,12 | 2,3,5,9,10 | 1,2,3,4,5 |  |
| 17 | Panama Gulf  of Chiriqui | 3,5,6,8,9,10,12 | 2,3,5,9,10 | 1,2,3,4,5 |  |
| 18 | Panama Bight | 3,5 | 3,5 presumably | 1,6,10 |  |
| 19 | Guayaquil | 3,4 presumably | 3,4 presumably | 2,3,7 |  |
| 20 | Cocos Islands | 2,5,6,8,9,10,12 | 2,3,5,9,10 | 1,2,3,4,5 |  |
| 21 | Northern Galapagos Islands | 3,4 presumably | 3,4 presumably | 2,3,7 |  |
| 22 | Western Galapagos Islands | 3,4 presumably | 3,4 presumably | 2,3,7 |  |
| 23 | Eastern Galapagos Islands | 3,4 presumably | 3,4 presumably | 2,3,7 |  |

**Supplementary Table S4.** Mean live coral cover and total larvae release for each ecoregion. The coral cover for Rapa-Pitcairn, Marquesas, Chiapas-Nicaragua and Guayaquil were mean values from the closest ecoregion. The coral cover for Hawaii was fixed at 25%. For each grid cell that is 100% occupied by a reef in the spatial domain, 1.0 units of larvae were released. Based on this ratio, the number of larvae released to each grid habitat cell was adjusted proportionally with the ecoregion mean live coral cover. Total larvae release can be transformed to numbers of larvae using the formula **r** * **a** * **P**, where **r** is the reproductive output (fecundity * abundance) per km^2^ of suitable habitat, **a** is the cell area in km^2^, and **P** is the connectivity probability matrix simulated^11^.

| **RegID** | **Ecoregion** | **Mean live coral cover (%)** | **Total release**  **(units)** | **References** |
| --- | --- | --- | --- | --- |
| 1 | Hawaii | 25.00 | 11.88735 |  |
| 2 | Hawaii | 25.00 | 4.10108 |  |
| 3 | Hawaii | 25.00 | 0.36497 |  |
| 4 | Line Islands | 35.00 | 2.55509 | \| Kenyon, et al. ^12^, Williams, et al. ^13^, Sandin, et al. ^14^ \| \| --- \| \|  \| |
| 5 | Tuamotus North | 23.00 | 0.20525 |  |
| 6 | Tuamotus Central | 23.00 | 25.00185 |  |
| 7 | Tuamotus South | 23.00 | 3.48549 | Adjeroud, et al. ^15^ |
| 8 | Rapa-Pitcairn | 23.00 | 0.24775 |  |
| 9 | Marquesas | 23.00 | 1.20821 |  |
| 10 | Easter Island | 48.00 | 0.40296 | Friedlander, et al. ^16^ |
| 11 | Desventuradas Islands | 48.00 | 0.09778 | Friedlander, et al. ^16^ |
| 12 | Cortezian (GoC) | 31.92 | 0.76296 | Reyes-Bonilla and Calderon-Aguilera ^17^, Robinson and Thomson ^18^, Aranceta-Garza, et al. ^19^ |
| 13 | Mexican Tropical Pacific | 32.96 | 0.36012 | Reyes-Bonilla, et al. ^20^, Pérez-Vivar, et al. ^21^ |
| 14 | Revillagigedos | 41.93 | 0.32224 | López-Pérez, et al. ^22^, Carriquiry and Reyes-Bonilla ^23^ |
| 15 | Clipperton | 53.23 | 0.09688 | Glynn, et al. ^24^ |
| 16 | Chiapas-Nicaragua | 30.00 | 0.02500 |  |
| 17 | Nicoya – Gulf of Chiriqui | 24.25 | 0.98215 | Eakin ^25^, Glynn ^26^, Glynn, et al. ^27^, Glynn ^28^, Glynn ^29^, Glynn, et al. ^30^, Glynn, et al. ^31^, Guzman and Robertson ^32^, Guzman, et al. ^33^, Guzman, et al. ^34^, Guzmán, et al. ^35^, Guzman, et al. ^36^, Jiménez and Cortés ^37^,Guzman, et al. ^34^ Glynn, et al. ^30^, Jiménez ^38^, Cortes ^39^ |
| 18 | Panama Bight | 29.19 | 1.33640 | Glynn, et al. ^40^, Guzman and Robertson ^32^ Guzmán, et al. ^35^, Glynn ^26^, Glynn, et al. ^30^, Garzón-Ferreira and Pinzón ^41^, Palacios, et al. ^42^, Glynn, et al. ^27^, Guzman Hector and Lopez Juan ^43^, Garzón-Ferreira and Rodríguez-Ramírez ^44^, Zapata, et al. ^45^, Vargas-Angel ^46^ |
| 19 | Guayaquil | 30.00 | 0.04722 |  |
| 20 | Cocos Islands | 21.1 | 0.04298 | Guzman and Robertson ^32^, Guzman and Cortes ^47^, Alvarado, et al. ^48^ |
| 21 | Northern Galapagos Islands | 11.38 | 0.00773 | Glynn and Wellington ^49^, Guzman and Robertson ^32^, Glynn, et al. ^50^, Glynn, et al. ^30^, Edgar, et al. ^51^ |
| 22 | Western Galapagos Islands | 11.38 | 0.00738 | Glynn and Wellington ^49^, Guzman and Robertson ^32^, Glynn, et al. ^50^, Glynn, et al. ^30^, Edgar, et al. ^51^ |
| 23 | Eastern Galapagos Islands | 11.38 | 0.36528 | Glynn and Wellington ^49^, Guzman and Robertson ^32^, Glynn, et al. ^50^, Glynn, et al. ^30^, Edgar, et al. ^51^ |

**Appendix 2**

**Model parameters and model output**

In the biophysical model, the biological parameters, currents velocity and turbulent diffusion controlled the overall dynamics of the cloud of larvae (See Treml *et al.*^11^ for detailed numerical methods and sensitivity testing). During each simulation and at each time step, the simulator tracked the cloud of larvae to record the position as it moves through the seascape. Larval competency and mortality determined the proportion of larvae settled in each habitat cells. If larvae encountered a suitable patch, the concentration of larvae settling with the habitat was recorded and generated a probability value. Ocean current velocities were interpolated from the HYCOM grid to the model spatial grid, using a cubic spline algorithm. A diffusivity parameter of 100 m^2^s^-1^ was used to represent sub-scale turbulence and mixing^11^.

We used as a framework a previous study^1^, yet our works differs in: (i) a larger spatial domain by including the Central Pacific. (ii) Three additional species were modeled (*Porites lobata, Pavona varians* and *Acropora valida*). (iii) The Central Pacific was modeled with a different reproductive phenology. (iv) The EP-Barrier and dispersal pulses are studied. (v) SST and ENSO intensity was correlated to connectivity pathways.

The genera *Pocillopora* spp. and *Porites* spp. have a cryptic taxonomy. We follow the new taxonomy for pocilloporids proposed by Schmidt-Roach^52^, in which *Pocillopora damicornis* is not distributed in the ETP. Species’ classification in the field based on the skeleton morphology is inaccurate due to high morphological plasticity^52,53^ and is likely that the reproductive phenology observations represent the species *Pocillopora meandrina* and *Pocillopora eydouxi*. Therefore, we refer to these observations as the *Pocillopora meandrina/eydouxi* complex. In the Eastern Tropical Pacific, the species *Porites lobata* and *Porites evermanni* also are indistinguishable by morphology in the field^54^, and are referred to as *Porites lobata/evermanni* complex.

The pelagic larval duration of the broadcast spawners *P. meandrina/eydouxi complex*, *P. lobata/evermanni*, and *Pavona varians* are unknown. We used two PLD values (100 and 150 d) for *P. meandrina/eydouxi complex.* The first PLD value used observations under controlled conditions of planula larvae of the brooder *P. damicornis*^55,56^. For the brooder *P. damicornis* also is available information on the pre-competency period of 3-10^57^ to 12 days^58^. The second value of 150 d PLD were supported in the large geographic range observed in the broadcast spawner pocilloporid^53^ (types 1 and 3) and three *Pocillopora* species (*P. meandrina*, *P. verrucosa,* and *P. eydouxi*) described by Schmidt-Roach *et al.*^52^. Also, supported in the first estimations of transport time to cross the EPB (e.g. 50 to 120 days^24,55,59,60^). The maximum PLD for any broadcast-spawning scleractinian corals can range 195 to 244 d^61^.

*Porites lobata* is a gonochoric broadcast spawner^62^. We used a 50 d PLD based on observations in Hawaii of Field^7^, who collected using containers *P. lobata* colonies and after spawning measured its larval viability for 53 days. *P. lobata* is the only large, massive poritid species in the ETP^62^. Its larvae contain zooxanthellae and can obtain nutrition during planktonic phases extending their dispersal potential^62^.

For *P. varians* we applied the shortest PLD of 30 d based on observations of Glynn *et al.*^63^ who describes that eggs were enveloped in mucus-laden strings that adhered to colony surfaces. They also described a larval pre-competency period 24-36 h, that swimming larva concentrated at the bottom of containers after 36 h, and can survive more than 10 days in containers.

We selected for *Acropora valida* a PLD of 120 d, based on its maximum pelagic larval duration of 100-130 days^64,65^.

**Model output**

To calculate a single adjacency connectivity probability matrix (**P**) and a single migration matrix (**M**) calculation, the cumulative probability of dispersal matrices (dispersal and density matrices) can be converted to numbers of larvae by multiplying their reproductive output per km^2^ of suitable habitat (Table S4). The entries of **P,** quantified the cumulative likelihood (connectivity strength) that a larva released from each ecoregion survives to settle on another. The diagonal of **P,** represented the cumulative probability of local retention or the proportion of larvae produced by a population that settles in the same population. The matrix **M** described the proportion of settlers at an ecoregion that came from a source ecoregion *i*. The diagonal of **M** is the cumulative proportion of self-recruitment, defined as the proportion of total settlers to a site that originated from that site ^11^. We transformed the matrix **M** to a biophysical distance matrix (**D**) using log (**M**^-1^). Distance matrix values are in the same rank-order as the geographic distance (i.e., a high proportion of settlers then have a short dispersal distance)^66^.

**Appendix 3**

**Regression analysis, connectivity metrics and network analysis**

**Supplementary Table S5** Results of linear regression analyses on the relationship between the probability of connectivity (response variable) and the ENSO intensity (predictor variable) from/to ecoregions. ENSO intensity is described as: Positive anomalies (El Niño), negative anomalies (La Niña), and all (positive and negatives). Significant results are in bold.

| **Analysis** | **ENSO intensity** | ***df*** | ***F*** | ***P*** | **_Adjusted_ R^2^** |
| --- | --- | --- | --- | --- | --- |
| **Line Islands-Clipperton Atoll dispersal route (~ 5,000 km)** | | | | | |
| From Line Islands to Clipperton Atoll | All | 6 | 0.537 | 0.491 | -0.070 |
| From Clipperton Atoll to Line Islands | All | 4 | 0.035 | 0.858 | -0.238 |
| **Galapagos Islands-Marquesas Islands dispersal route (~ 4,900 km)** | | | | | |
| From Northern Galapagos Islands to Marquesas Islands | All | 88 | 0.166 | 0.684 | -0.009 |
| From Northern Galapagos Islands to Marquesas Islands | Positive | 31 | 1.011 | 0.322 | 0.001 |
| From Northern Galapagos Islands to Marquesas Islands | Negative | 55 | 2.949 | 0.091 | 0.033 |
| From Eastern Galapagos Islands to Marquesas Islands | All | 331 | 0.005 | 0.938 | -0.003 |
| From Eastern Galapagos Islands to Marquesas Islands | Positive | 137 | 1.067 | 0.303 | 0.001 |
| From Eastern Galapagos Islands to Marquesas Islands | Negative | 190 | 1.103 | 0.295 | 0.001 |
| **Clipperton Atoll-Hawaii Central dispersal route (~ 5,100 km)** | | | | | |
| From Clipperton Atoll to Hawaii Central | All | 11 | 0.049 | 0.828 | -0.086 |
| From Clipperton Atoll to Hawaii Central | Negative | 7 | 0.041 | 0.844 | -0.136 |
| **Closed loop and semiclosed loop** | | | | | |
| From Panama Bight to Nicoya | **All** | **407** | **51.9** | <**0.001** | **0.111** |
| From Panama Bight to Nicoya | Positive | 184 | 0.844 | 0.359 | 0.001 |
| From Panama Bight to Nicoya | **Negative** | **219** | **23.97** | **<0.001** | **0.094** |
| From Panama Bight to Northern Galapagos Islands | **All** | **111** | **38.33** | **<0.001** | **0.250** |
| From Panama Bight to Northern Galapagos Islands | **Positive** | **52** | **15.71** | **<0.001** | **0.217** |
| From Panama Bight to Northern Galapagos Islands | **Negative** | **57** | **13.86** | **<0.001** | **0.181** |
| From Panama Bight to Eastern Galapagos Islands | All | 143 | 0.182 | 0.669 | -0.005 |
| From Panama Bight to Eastern Galapagos Islands | Positive | 64 | 2.617 | 0.110 | 0.024 |
| From Panama Bight to Eastern Galapagos Islands | Negative | 77 | 5.887 | 0.017 | 0.058 |
| From Guayaquil to Panama Bight | All | **111** | **38.33** | **<0.001** | **0.250** |
| From Guayaquil to Panama Bight | Positive | **52** | **15.71** | **<0.001** | **0.217** |
| From Guayaquil to Panama Bight | Negative | **57** | **13.86** | **<0.001** | **0.180** |

**Supplementary Table S6.** Connectivity metrics used to represent ecoregional-scale and conservation value of spatially structured populations.

| **Measure** | **Description** |
| --- | --- |
| **Degree** | Out-degree or the number of outcoming connections from a particular ecoregion (node) to other ecoregions. In-degree represents the number of incoming connections to an ecoregion |
| **Connectivity loops** | A loop is defined as a closed path from a node to itself (i.e. begins and ends at the same node)^67^. In a connectivity matrix, as successive generations occur, the dispersal of larvae flows around the loop and therefore influences its future dynamics (e.g. from population A to B, from B to C, from C to A). Loops are critical for metapopulation persistence^68^ |
| **Betweenness centrality** | The flow that an ecoregion can control regarding their position in a network^69^. Captures the most used paths between all pairs of ecoregions in the network, and can be used to identify important stepping-stones, dispersal pathways^70^ or stepping-stone migration distance^71^ |
| **Closeness centrality** | Indicates how close, or the length of the average shortest path, between an ecoregion and all other ecoregions in the network. Values are higher for a particular ecoregion when its total distance to all other ecoregions in the network is lower^72^ |
| **Local retention and ecoregional retention** | Local retention is the proportion of larvae produced by a population that recruits into the natal population^68^. A subpopulation is more likely to be self-sustaining with higher values of local retention^66^. This term refers to a patch or local reef.  Analogously, here we defined ecoregional retention as the proportion of larvae produced by a group of populations that recruits into the same ecoregion. Also, the subpopulation within the ecoregion is more likely to be self-sustaining with higher values of ecoregional retention. Ecoregional scale – refers to a group of reefs within similar species composition and particular oceanographic or topographic features, clearly distinct from adjacent ecoregions^73^. Ecoregion instead of the local reef was selected to answer broad-scale questions of long-distance dispersal. |
| **Self- recruitment** | Proportion of recruitment to a site/ecoregion comprised of larvae born on that site/ecoregion^68^ |

**Supplementary Table S7**. Connectivity and network analysis for the Maximum dispersal potential model and reef-building species. Codes correspond to: Hawaii North (1), Hawaii Central (2), Hawaii South (3), Line Islands (4), Tuamotus North (5), Tuamotus Central (6), Tuamotus South (7), Rapa-Pitcairn (8), Marquesas Islands (9), Easter Island (10), Desventuradas Islands (11), Cortezian (GoC) (12), Mexican Tropical Pacific (13), Revillagigedos (14), Clipperton (15), Chiapas-Nicaragua (16), Nicoya (17), Panama Bight (18), Guayaquil (19), Cocos Islands (20), Northern Galapagos Islands (21), Western Galapagos Islands (22) and Eastern Galapagos Islands (23).

| **Maximum dispersal potential model** | **1** | **2** | **3** | **4** | **5** | **6** | **7** | **8** | **9** | **10** | **11** | **12** | **13** | **14** | **15** | **16** | **17** | **18** | **19** | **20** | **21** | **22** | **23** |
| --- | --- | --- | --- | --- | --- | --- | --- | --- | --- | --- | --- | --- | --- | --- | --- | --- | --- | --- | --- | --- | --- | --- | --- |
| **In-degree** | 2 | 3 | 3 | 6 | 6 | 5 | 3 | 2 | 6 | 1 | 0 | 4 | 10 | 4 | 12 | 9 | 10 | 9 | 5 | 11 | 7 | 6 | 7 |
| **Out-degree** | 2 | 2 | 2 | 4 | 2 | 4 | 2 | 2 | 4 | 0 | 0 | 4 | 7 | 5 | 11 | 5 | 12 | 10 | 9 | 9 | 12 | 10 | 13 |
| **Betweenness** | 7 | 12 | 12 | 110 | 51 | 81 | 51 | 17 | 41 | 0 | 0 | 3 | 10 | 0 | 141 | 0 | 35 | 30 | 0 | 25 | 2 | 0 | 66 |
| **Closeness** | 0.00261 | 0.00282 | 0.00312 | 0.00424 | 0.00438 | 0.00399 | 0.00364 | 0.00336 | 0.00465 | 0.00212 | 0.00198 | 0.00364 | 0.00384 | 0.00374 | 0.00468 | 0.00447 | 0.00467 | 0.00491 | 0.00348 | 0.00492 | 0.00510 | 0.00513 | 0.00517 |
| **Ecoregional retention** | 1.00000 | 1.00000 | 0.00000 | 0.99999 | 0.29747 | 1.00000 | 1.00000 | 0.88906 | 1.00000 | 1.00000 | 0.74338 | 1.00000 | 1.00000 | 1.00000 | 0.25166 | 0.99641 | 1.00000 | 1.00000 | 0.82811 | 0.13657 | 0.00411 | 0.57428 | 0.97602 |
| **Self-recruitment** | 0.75073 | 0.94624 | 0.96149 | 0.99435 | 0.56332 | 0.86979 | 0.77074 | 0.71610 | 0.99702 | 0.99984 | 1.00000 | 0.99104 | 0.75158 | 0.78758 | 0.88667 | 0.36664 | 0.78526 | 0.95572 | 0.99049 | 0.28234 | 0.02215 | 0.07589 | 0.97620 |
|  |  |  |  |  |  |  |  |  |  |  |  |  |  |  |  |  |  |  |  |  |  |  |  |
| ***Pocillopora PLD150 model*** | **1** | **2** | **3** | **4** | **5** | **6** | **7** | **8** | **9** | **10** | **11** | **12** | **13** | **14** | **15** | **16** | **17** | **18** | **19** | **20** | **21** | **22** | **23** |
| **In-degree** | 2 | 1 | 0 | 2 | 1 | 3 | 2 | 1 | 0 | 0 | 0 | 1 | 2 | 1 | 7 | 1 | 2 | 3 | 1 | 6 | 5 | 3 | 5 |
| **Out-degree** | 1 | 1 | 1 | 0 | 2 | 1 | 2 | 1 | 3 | 0 | 0 | 3 | 2 | 1 | 0 | 0 | 6 | 4 | 5 | 5 | 4 | 4 | 3 |
| **Betweenness** | 1 | 0 | 0 | 0 | 0 | 4 | 4 | 0 | 0 | 0 | 0 | 8 | 14 | 0 | 0 | 0 | 24 | 5 | 0 | 24 | 5 | 0 | 3 |
| **Closeness** | 0.00210 | 0.00208 | 0.00204 | 0.00203 | 0.00219 | 0.00224 | 0.00218 | 0.00211 | 0.00224 | 0.00198 | 0.00198 | 0.00231 | 0.00237 | 0.00240 | 0.00273 | 0.00245 | 0.00279 | 0.00273 | 0.00240 | 0.00282 | 0.00285 | 0.00284 | 0.00285 |
| **Ecoregional retention** | 0.30105 | 0.59566 | 0.03461 | 0.38449 | 0.00811 | 0.56875 | 0.37518 | 0.06553 | 0.39676 | 0.60273 | 0.04530 | 0.40532 | 0.36677 | 0.37020 | 0.00224 | 0.20004 | 0.47221 | 0.63509 | 0.05081 | 0.00667 | 0.00002 | 0.02626 | 0.13352 |
| **Self-recruitment** | 0.98959 | 0.99964 | 1.00000 | 0.99999 | 0.94167 | 0.99816 | 0.99624 | 0.98183 | 1.00000 | 1.00000 | 1.00000 | 0.99998 | 0.99535 | 0.99979 | 0.99554 | 0.99214 | 0.99828 | 0.99993 | 0.99999 | 0.94592 | 0.01011 | 0.13926 | 0.99891 |
|  |  |  |  |  |  |  |  |  |  |  |  |  |  |  |  |  |  |  |  |  |  |  |  |
| ***Pocillopora PLD100 model*** | **1** | **2** | **3** | **4** | **5** | **6** | **7** | **8** | **9** | **10** | **11** | **12** | **13** | **14** | **15** | **16** | **17** | **18** | **19** | **20** | **21** | **22** | **23** |
| **In-degree** | 2 | 1 | 0 | 2 | 1 | 3 | 2 | 1 | 0 | 0 | 0 | 1 | 2 | 1 | 7 | 1 | 2 | 3 | 1 | 6 | 5 | 3 | 5 |
| **Out-degree** | 1 | 1 | 1 | 0 | 2 | 1 | 2 | 1 | 3 | 0 | 0 | 3 | 2 | 1 | 0 | 0 | 6 | 4 | 5 | 5 | 4 | 4 | 3 |
| **Betweenness** | 1 | 0 | 0 | 0 | 0 | 4 | 4 | 0 | 0 | 0 | 0 | 8 | 14 | 0 | 0 | 0 | 24 | 5 | 0 | 24 | 5 | 0 | 3 |
| **Closeness** | 0.00210 | 0.00208 | 0.00204 | 0.00203 | 0.00219 | 0.00224 | 0.00218 | 0.00211 | 0.00224 | 0.00198 | 0.00198 | 0.00231 | 0.00237 | 0.00240 | 0.00273 | 0.00245 | 0.00279 | 0.00273 | 0.00240 | 0.00282 | 0.00285 | 0.00284 | 0.00285 |
| **Ecoregional retention** | 0.30105 | 0.59566 | 0.03461 | 0.38449 | 0.00811 | 0.56875 | 0.37518 | 0.06553 | 0.39676 | 0.60273 | 0.04530 | 0.40532 | 0.36677 | 0.37020 | 0.00224 | 0.20004 | 0.47221 | 0.63509 | 0.05081 | 0.00667 | 0.00002 | 0.02626 | 0.13352 |
| **Self-recruitment** | 0.98959 | 0.99964 | 1.00000 | 0.99999 | 0.94167 | 0.99816 | 0.99624 | 0.98184 | 1.00000 | 1.00000 | 1.00000 | 0.99998 | 0.99535 | 0.99979 | 0.99554 | 0.99214 | 0.99828 | 0.99993 | 0.99999 | 0.94592 | 0.01011 | 0.13926 | 0.99891 |

|  |  |  |  |  |  |  |  |  |  |  |  |  |  |  |  |  |  |  |  |  |  |  |  |
| --- | --- | --- | --- | --- | --- | --- | --- | --- | --- | --- | --- | --- | --- | --- | --- | --- | --- | --- | --- | --- | --- | --- | --- |
| ***Porites model*** | **1** | **2** | **3** | **4** | **5** | **6** | **7** | **8** | **9** | **10** | **11** | **12** | **13** | **14** | **15** | **16** | **17** | **18** | **19** | **20** | **21** | **22** | **23** |
| **In-degree** | 2 | 1 | 0 | 1 | 1 | 3 | 2 | 1 | 0 | 0 | 0 | 1 | 2 | 1 | 3 | 1 | 2 | 3 | 0 | 5 | 5 | 4 | 5 |
| **Out-degree** | 1 | 1 | 1 | 0 | 2 | 1 | 2 | 1 | 2 | 0 | 0 | 2 | 2 | 1 | 0 | 0 | 5 | 5 | 4 | 3 | 4 | 3 | 3 |
| **Betweenness** | 1 | 0 | 0 | 0 | 1 | 4 | 4 | 0 | 0 | 0 | 0 | 8 | 16 | 0 | 0 | 0 | 24 | 5 | 0 | 19 | 8 | 0 | 5 |
| **Closeness** | 0.00209 | 0.00207 | 0.00203 | 0.00197 | 0.00219 | 0.00222 | 0.00216 | 0.00209 | 0.00222 | 0.00198 | 0.00198 | 0.00210 | 0.00232 | 0.00216 | 0.00240 | 0.00232 | 0.00259 | 0.00265 | 0.00230 | 0.00269 | 0.00273 | 0.00271 | 0.00272 |
| **Ecoregional retention** | 0.37158 | 0.65565 | 0.06877 | 0.43986 | 0.00948 | 0.63226 | 0.44199 | 0.08308 | 0.46247 | 0.69502 | 0.05154 | 0.47185 | 0.42723` | 0.44900 | 0.00259 | 0.23672 | 0.53941 | 0.71258 | 0.06575 | 0.01006 | 0.00002 | 0.03290 | 0.15773 |
| **Self-recruitment** | 0.98818 | 0.99969 | 1.00000 | 1.00000 | 0.95140 | 0.99784 | 0.99634 | 0.98407 | 1.00000 | 1.00000 | 1.00000 | 0.99998 | 0.99599 | 0.99983 | 0.99838 | 0.98905 | 0.99889 | 0.99993 | 1.00000 | 0.96519 | 0.00781 | 0.15252 | 0.99820 |
|  |  |  |  |  |  |  |  |  |  |  |  |  |  |  |  |  |  |  |  |  |  |  |  |
| ***P. varians*** | **1** | **2** | **3** | **4** | **5** | **6** | **7** | **8** | **9** | **10** | **11** | **12** | **13** | **14** | **15** | **16** | **17** | **18** | **19** | **20** | **21** | **22** | **23** |
| **In-degree** | 1 | 1 | 0 | 1 | 2 | 3 | 2 | 1 | 0 | 0 | 0 | 1 | 2 | 1 | 0 | 1 | 2 | 3 | 0 | 3 | 3 | 1 | 3 |
| **Out-degree** | 1 | 1 | 0 | 1 | 2 | 1 | 2 | 1 | 2 | 0 | 0 | 2 | 1 | 0 | 0 | 0 | 4 | 3 | 3 | 2 | 1 | 2 | 2 |
| **Betweenness** | 0 | 0 | 0 | 0 | 4 | 6 | 5 | 0 | 0 | 0 | 0 | 8 | 14 | 0 | 0 | 0 | 24 | 14 | 0 | 20 | 14 | 0 | 8 |
| **Closeness** | 0.00205 | 0.00205 | 0.00198 | 0.00211 | 0.00220 | 0.00225 | 0.00219 | 0.00205 | 0.00215 | 0.00198 | 0.00198 | 0.00199 | 0.00215 | 0.00173 | 0.00198 | 0.00232 | 0.00247 | 0.00243 | 0.00208 | 0.00249 | 0.00227 | 0.00226 | 0.00227 |
| **Ecoregional retention** | 0.53466 | 0.79243 | 0.03729 | 0.54495 | 0.01990 | 0.74164 | 0.46404 | 0.11104 | 0.59977 | 0.81995 | 0.06551 | 0.59387 | 0.53567 | 0.54138 | 0.01819 | 0.24421 | 0.62754 | 0.83964 | 0.09938 | 0.00559 | 0.00025 | 0.05725 | 0.20178 |
| **Self-recruitment** | 0.99244 | 0.99992 | 1.00000 | 1.00000 | 0.98872 | 0.99711 | 0.99683 | 0.99954 | 1.00000 | 1.00000 | 1.00000 | 0.99992 | 0.99358 | 0.99979 | 1.00000 | 0.94333 | 0.99872 | 0.99996 | 1.00000 | 0.88643 | 0.04343 | 0.15731 | 0.99885 |
|  |  |  |  |  |  |  |  |  |  |  |  |  |  |  |  |  |  |  |  |  |  |  |  |
| ***A. valida*** | **1** | **2** | **3** | **4** | **5** | **6** | **7** | **8** | **9** | **10** | **11** | **12** | **13** | **14** | **15** | **16** | **17** | **18** | **19** | **20** | **21** | **22** | **23** |
| **In-degree** | 1 | 1 | 0 | 1 | 2 | 3 | 2 | 1 | 0 | 0 | 0 | 0 | 0 |  |  |  |  |  |  |  |  |  |  |
| **Out-degree** | 1 | 1 | 0 | 1 | 2 | 1 | 2 | 1 | 2 | 0 | 0 | 0 | 0 |  |  |  |  |  |  |  |  |  |  |
| **Betweenness** | 0 | 0 | 0 | 0 | 4 | 6 | 5 | 0 | 0 | 0 | 0 | 0 | 0 |  |  |  |  |  |  |  |  |  |  |
| **Closeness** | 0.00205 | 0.00205 | 0.00198 | 0.00211 | 0.00220 | 0.00225 | 0.00219 | 0.00205 | 0.00215 | 0.00198 | 0.00198 | 0.00198 | 0.00198 |  |  |  |  |  |  |  |  |  |  |
| **Ecoregional retention** | 0.53466 | 0.79243 | 0.03729 | 0.54495 | 0.01990 | 0.74164 | 0.46404 | 0.11104 | 0.59977 | 0.81995 | 0.06551 | 0.00000 | 0.00000 |  |  |  |  |  |  |  |  |  |  |
| **Self-recruitment** | 0.99244 | 0.99992 | 1.00000 | 1.00000 | 0.98872 | 0.99711 | 0.99683 | 0.99954 | 1.00000 | 1.00000 | 1.00000 | 0.00000 | 0.00000 |  |  |  |  |  |  |  |  |  |  |

Bibliography

1 Romero-Torres, M., Acosta, A. & Treml, E. The regional structure of spawning phenology and the potential consequences for connectivity of coral assemblages across the Eastern Tropical Pacific. *ICES. J. Mar. Sci.* **74**, 613-624, doi:10.1093/icesjms/fsw218 (2017).

2 Kenyon, J. C. *Acropora* (Anthozoa: Scleractinia) Reproductive Synchrony and Spawning Phenology in the Northern Line Islands, Central Pacific, as Inferred from Size Classes of Developing Oocytes1. *Pac. Sci.* **62**, 569-578, doi:10.2984/1534-6188(2008)62[569:aasrsa]2.0.co;2 (2008).

3 Carroll, A., Harrison, P. & Adjeroud, M. Sexual reproduction of *Acropora* reef corals at Moorea, French Polynesia. *Coral Reefs* **25**, 93-97, doi:10.1007/s00338-005-0057-6 (2006).

4 Stimson, J. S. Mode and timing of reproduction in some common hermatypic corals of Hawaii and Enewetak. *Mar. Biol.* **48**, 173-184, doi:10.1007/BF00395017 (1978).

5 Richmond, R. H. & Jokiel, P. L. Lunar periodicity in larval release in the reef coral Pocillopora damicornis at Enewetak and Hawaii. *Bull. Mar. Sci.* **34**, 280-287 (1984).

6 Neves, E. G. Histological analysis of reproductive trends of three Porites species from Kane'ohe Bay, Hawai'i. *Pac. Sci.* **54**, 195-200 (2000).

7 Field, S. Settlement biology of larvae of *Montipora verrucosa* and *Porites lobata* in Hawaii. Reproduction in Reef Corals. 111-119 (1998).

8 Mate T, J. L. New reports on the timing and mode of reproduction of Hawaiian corals. 7 (1998).

9 Fiene-Severns, P. A note on synchronous spawning in the reef coral *Pocillopora meandrina* at Molokini Islet, Hawai‘i. 22-24 (1998).

10 Kenyon, J. C. Sexual reproduction in Hawaiian *Acropora*. *Coral Reefs* **11**, 37-43, doi:10.1007/BF00291933 (1992).

11 Treml, E. A. *et al.* Reproductive output and duration of the pelagic larval stage determine seascape-wide connectivity of marine populations. *Integr. Comp. Biol.* **52**, 525-537, doi:10.1029/2008JC005166 (2012).

12 Kenyon, J. C., Maragos, J. E. & Wilkinson, C. B. Characterization of coral communities at kingman reef in the remote central Pacific Ocean. *Atoll Res. Bull.* **584**, 1-29 (2010).

13 Williams, G. J. *et al.* Benthic communities at two remote Pacific coral reefs: effects of reef habitat, depth, and wave energy gradients on spatial patterns. *PeerJ* **1**, e81, doi:10.7717/peerj.81 (2013).

14 Sandin, S. A. *et al.* Baselines and degradation of coral reefs in the Northern Line Islands. *PLoS. ONE* **3**, doi:10.1371/journal.pone.0001548 (2008).

15 Adjeroud, M. *et al.* Recurrent disturbances, recovery trajectories, and resilience of coral assemblages on a South Central Pacific reef. *Coral Reefs* **28**, 775-780, doi:10.1007/s00338-009-0515-7 (2009).

16 Friedlander, A. M. *et al.* Effects of isolation and fishing on the marine ecosystems of Easter Island and Salas y Gómez, Chile. *Aquat. Conserv.: Mar. Freshw. Ecosyst.* **23**, 515-531, doi:10.1002/aqc.2333 (2013).

17 Reyes-Bonilla, H. & Calderon-Aguilera, L. E. Population density, distribution and consumption rates of three corallivores at Cabo Pulmo reef, Gulf of California, Mexico. *Mar. Ecol.* **20**, 347-357, doi:10.1046/j.1439-0485.1999.2034080.x (1999).

18 Robinson, J. A. & Thomson, D. A. Status of the Pulmo coral reefs in the lower Gulf of California. *Environ. Conserv.* **19**, 261-264, doi:10.1017/S0376892900031076 (1992).

19 Aranceta-Garza, F., Balart, E. F., Reyes-Bonilla, H. & Cruz-Hernández, P. Effect of tropical storms on sexual and asexual reproduction in coral *Pocillopora verrucosa* subpopulations in the Gulf of California. *Coral Reefs* **31**, 1157-1167, doi:10.1007/s00338-012-0941-9 (2012).

20 Reyes-Bonilla, H., Escobosa-González, L. E., Cupul-Magaña, A. L., Medina-Rosas, P. & Calderón-Aguilera, L. E. Community structure of zooxanthellate corals (Anthozoa: Scleractinia) in Carrizales coral reef, Pacific coast, Mexico. *Rev. Biol. Trop.* **61**, 583-594 (2013).

21 Pérez-Vivar, T. L., Reyes-Bonilla, H. & Padilla, C. Stony corals (Scleractinia) from the Marías Islands, Mexican Pacific. *Cienc. Mar.* **32**, 259-270, doi:10.7773/cm.v32i21.1063 (2006).

22 López-Pérez, R. A., Mora-Pérez, M. G. & Leyte-Morales, G. E. Coral (Anthozoa: Scleractinia) recruitment at Bahías de Huatulco, western México: Implications for coral community structure and dynamics. *Pac. Sci.* **61**, 355-369, doi:10.2984/1534-6188(2007)61[355:CASRAB]2.0.CO;2 (2007).

23 Carriquiry, J. D. & Reyes-Bonilla, H. Community structure and geographic distribution of the coral reefs of Nayarit, Mexican Pacific. *Cienc. Mar.* **23**, 227-248, doi:10.7773/cm.v23i2.793 (1997).

24 Glynn, P. W., Veron, J. E. N. & Wellington, G. M. Clipperton Atoll (eastern Pacific): oceanography, geomorphology, reef-building coral ecology and biogeography. *Coral Reefs* **15**, 71-99, doi:10.1007/BF01771897 (1996).

25 Eakin, C. M. Where have all the carbonates gone? A model comparison of calcium carbonate budgets before and after the 1982-1983 El Niño at Uva Island in the eastern Pacific. *Coral Reefs* **15**, 109-119, doi:10.1007/BF01771900 (1996).

26 Glynn, P. W. Some Physical and Biological Determinants of Coral Community Structure in the Eastern Pacific. *Ecol. Monogr.* **46**, 431-456, doi:10.2307/1942565 (1976).

27 Glynn, P. W., von Prahl, H. & Guhl, F. Coral reefs of Gorgona Island, Colombia, with special reference to corallivores and their influence on community structure and reef development. *An. Inst. Inv. Mar.– Punta de Betín.*, 185-214 (1982).

28 Glynn, P. W. Widespread coral mortality and the 1982-83 El Niño warming event. *Environ. Conserv.* **11**, 133-146, doi:10.1017/S0376892900013825 (1984).

29 Glynn, P. in *Proc 5th Int Coral Reef Symp.* 183-188.

30 Glynn, P. W., Cortés-Núñez, J., Guzmán-Espinal, H. M. & Richmond, R. in *Proceedings of the 6th International Coral Reef Symposium, Australia.* 237-243.

31 Glynn, P. W., Enochs, I. C., Afflerbach, J. A., Brandtneris, V. W. & Serafy, J. E. Eastern Pacific reef fish responses to coral recovery following El Niño disturbances. *Mar. Ecol. Prog. Ser.* **495**, 233-247, doi:10.3354/meps10594 (2014).

32 Guzman, H. M. & Robertson, D. R. Population and feeding responses of the corallivorous pufferfish Arothron meleagris to coral mortality in the eastern Pacific. *Mar. Ecol. Prog. Ser.* **55**, 121-131 (1989).

33 Guzman, H. M., Guevara, C. A. & Breedy, O. Distribution, diversity, and conservation of coral reefs and coral communities in the largest marine protected area of Pacific Panama (Coiba Island). *Environ. Conserv.* **31**, 111-121, doi:10.1017/s0376892904001250 (2004).

34 Guzman, H. M., Cortes, J., Glynn, P. W. & Richmond, R. H. Coral mortality associated with dinoflagellate blooms in the eastern Pacific (Costa Rica and Panama). *Mar. Ecol. Prog. Ser.* **60**, 299-303 (1990).

35 Guzmán, H. M., Robertson, D. R. & Díaz, M. L. Distribución y abundancia de corales en el arrecife del Refugio de Isla Iguana, Pacífico de Panamá. *Rev. Biol. Trop.* **39**, 225-231 (1991).

36 Guzman, H. M., Cortes, J., Richmond, R. H. & Glynn, P. W. Effects of "El Niño - Southern oscillation' 1982/83 in the coral reefs at Isla del Caño, Costa Rica. *Rev. Biol. Trop.* **35**, 325-332 (1987).

37 Jiménez, C. E. & Cortés, J. Coral cover change associated to El Niño, Eastern Pacific, Costa Rica, 1992-2001. *Mar. Ecol.* **24**, 179-192, doi:10.1046/j.1439-0485.2003.03814.x (2003).

38 Jiménez, C. Arrecifes y ambientes coralinos de Bahía Culebra, Pacífico de Costa Rica: aspectos biológicos, económico-recreativos y de manejo. *Rev. Biol. Trop.* **49**, 215-231 (2001).

39 Cortes, J. The coral reefs of Golfo Dulce, Costa Rica: distribution and community structure. *Atoll Res. Bull.* **344**, 1-37 (1990).

40 Glynn, P. W., Stewart, R. H. & McCosker, J. E. Pacific coral reefs of Panamá: Structure, distribution and predators. *Geol. Rundsch.* **61**, 483-519, doi:10.1007/bf01896330 (1972).

41 Garzón-Ferreira, J. & Pinzón, J. Evaluación rápida de estructura y salud de las formaciones coralinas de la Isla de Malpelo (Pacífico colombiano). *Bol. Invest. Mar. Costeras.* **28**, 137-154 (1999).

42 Palacios, M. M., Muñoz, C. G. & Zapata, F. A. Fish corallivory on a pocilloporid reef and experimental coral responses to predation. *Coral Reefs* **33**, 625-636, doi:10.1007/s00338-014-1173-y (2014).

43 Guzman Hector, M. & Lopez Juan, D. Diet of the corallivorous pufferfish *Arothron meleagris* (Pisces: Tetradontidae) at Gorgona Island, Colombia. *Rev. Biol. Trop.* **39 (2)**, 203-206 (1991).

44 Garzón-Ferreira, J. & Rodríguez-Ramírez, A. Estado de los arrecifes coralinos en colombia año 2000. 12 (2000).

45 Zapata, F. A., Rodríguez-Ramírez, A., Caro-Zambrano, C. & Garzón-Ferreira, J. Mid-term coral-algal dynamics and conservation status of a Gorgona Island (Tropical Eastern Pacific) coral reef. *Rev. Biol. Trop.* **58**, 81-94 (2010).

46 Vargas-Angel, B. Distribution and community structure of the reef corals of Ensenada de Utría, Pacific coast of Colombia. *Rev. Biol. Trop.* **44**, 643-651 (1996).

47 Guzman, H. M. & Cortes, J. Reef recovery 20 years after the 1982-1983 El Niño massive mortality. *Mar. Biol.* **151**, 401-411, doi:10.1007/s00227-006-0495-x (2007).

48 Alvarado, J. J., Cortés, J. & Reyes-Bonilla, H. Reconstruction of *Diadema mexicanum* bioerosion impact on three Costa Rican Pacific coral reefs. *Rev. Biol. Trop.* **60**, 121-132 (2012).

49 Glynn, P. W. & Wellington, G. M. *Corals and Coral Reefs of the Galápagos Islands*. (University of California Press, 1983).

50 Glynn, P. W., Riegl, B., Correa, A. M. & Baums, I. B. Rapid recovery of a coral reef at Darwin Island, Galapagos Islands. *Galapagos. Res.* **66**, 6-13 (2009).

51 Edgar, G. J. *et al.* El Niño, grazers and fisheries interact to greatly elevate extinction risk for Galápagos marine species. *Glob. Change. Biol.* **16**, 2876-2890, doi:10.1111/j.1365-2486.2009.02117.x (2010).

52 Schmidt-Roach, S., Miller, K. J., Lundgren, P. & Andreakis, N. With eyes wide open: a revision of species within and closely related to the *Pocillopora damicornis* species complex (Scleractinia; Pocilloporidae) using morphology and genetics. *Zool. J. Linn. Soc.* **170**, 1-33, doi:10.1111/zoj.12092 (2014).

53 Pinzón, J. H. *et al.* Blind to morphology: genetics identifies several widespread ecologically common species and few endemics among Indo-Pacific cauliflower corals (*Pocillopora*, Scleractinia). *J. Biogeogr.* **40**, 1595–1608, doi:10.1111/jbi.12110 (2013).

54 Hellberg, M. E., Prada, C., Tan, M. H., Forsman, Z. H. & Baums, I. B. Getting a grip at the edge: Recolonization and introgression in eastern Pacific *Porites* corals. *J. Biogeogr.* **43**, 2147–2159, doi:10.1111/jbi.12792 (2016).

55 Richmond, R. H. Energetics, competency, and long-distance dispersal of planula larvae of the coral *Pocillopora damicornis*. *Mar. Biol.* **93**, 527-533, doi:10.1007/BF00392790 (1987).

56 Harii, S., Kayanne, H., Takigawa, H., Hayashibara, T. & Yamamoto, M. Larval survivorship, competency periods and settlement of two brooding corals, *Heliopora* coerulea and *Pocillopora damicornis*. *Mar. Biol.* **141**, 39-46, doi:10.1007/s00227-002-0812-y (2002).

57 Lee, C. S., Walford, J. & Goh, B. P. L. Adding coral rubble to substrata enhances settlement of Pocillopora damicornis larvae. *Coral Reefs* **28**, 529-533, doi:10.1007/s00338-009-0467-y (2009).

58 Isomura, N. & Nishihira, M. Size variation of planulae and its effect on the lifetime of planulae in three pocilloporid corals. *Coral Reefs* **20**, 309-315, doi:10.1007/s003380100180 (2001).

59 Grigg, R. W. & Hey, R. Paleoceanography of the tropical Eastern Pacific Ocean. *Science* **255**, 172-178, doi:10.1126/science.255.5041.172 (1992).

60 Leis, J. M. Larval fish dispersal and the East Pacific Barrier. *Oceanogr. Trop.* **19**, 181-192 (1984).

61 Graham, E. M., Baird, A. H. & Connolly, S. R. Survival dynamics of scleractinian coral larvae and implications for dispersal. *Coral Reefs* **27**, 529-539, doi:10.1007/s00338-008-0361-z (2008).

62 Glynn, P. W. *et al.* Reef coral reproduction in the eastern Pacific: Costa Rica, Panama, and Galapagos Islands (Ecuador). 2. Poritidae. *Mar. Biol.* **118**, 191-208, doi:10.1007/BF00349785 (1994).

63 Glynn, P. W., Colley, S. B., Ting, J. H., Mate, J. L. & Guzman, H. M. Reef coral reproduction in the eastern Pacific: Costa Rica, Panama and Galapagos Islands (Ecuador). 4. Agariciidae, recruitment and recovery of *Pavona varians* and *Pavona* sp.a. *Mar. Biol.* **136**, 785-805, doi:10.1007/s002270000286 (2000).

64 Baird, A. H. *The ecology of coral larvae: settlement patterns, habitat selection and the length of the larval phase* Doctor of Philosophy in Marine Ecology thesis, James Cook University, (2001).

65 Connolly, S. R. & Baird, A. H. Estimating dispersal potential for marine larvae: dynamic models applied to scleractinian corals. *Ecology* **91**, 3572-3583, doi:10.1890/10-0143.1 (2010).

66 Schill, S. R. *et al.* No Reef Is an Island: Integrating Coral Reef Connectivity Data into the Design of Regional-Scale Marine Protected Area Networks. *PLoS. ONE* **10**, e0144199, doi:10.1371/journal.pone.0144199 (2015).

67 Caswell, H. *Matrix population models: construction, analysis, and interpretation*. 2nd edn, (Sinauer Associates, 2001).

68 Burgess, S. C. *et al.* Beyond connectivity: How empirical methods can quantify population persistence to improve marine protected-area design. *Ecol. Appl.* **24**, 257-270, doi:10.1890/13-0710.1 (2014).

69 Börner, K., Sanyal, S. & Vespignani, A. Network science. *Annu. Rev. Inform. Sci.* **41**, 537-607, doi:10.1002/aris.2007.1440410119 (2007).

70 Treml, E. A., Halpin, P. N., Urban, D. L. & Pratson, L. F. Modeling population connectivity by ocean currents, a graph-theoretic approach for marine conservation. *Landsc. Ecol.* **23**, 19-36, doi:10.1007/s10980-007-9138-y (2008).

71 Liggins, L., Treml, E. A., Possingham, H. P. & Riginos, C. Seascape features, rather than dispersal traits, predict spatial genetic patterns in co-distributed reef fishes. *J. Biogeogr.* **43**, 256-267, doi:10.1111/jbi.12647 (2016).

72 Beger, M. *et al.* Integrating regional conservation priorities for multiple objectives into national policy. *Nat. Commun.* **6**, 8208, doi:10.1038/ncomms9208 (2015).

73 Spalding, M. D. *et al.* Marine Ecoregions of the World: A Bioregionalization of Coastal and Shelf Areas. *Bioscience* **57**, 573-583, doi:10.1641/b570707 (2007).
